# Substrate Specificity of T7 RNA Polymerase toward Hypophosphoric Analogues of ATP

**DOI:** 10.1021/acsomega.3c08635

**Published:** 2024-02-13

**Authors:** Roza Pawlowska, Anna Graczyk, Ewa Radzikowska-Cieciura, Ewelina Wielgus, Rafal Madaj, Arkadiusz Chworos

**Affiliations:** † Department of Bioorganic Chemistry, Centre of Molecular and Macromolecular Studies, Polish Academy of Sciences, Sienkiewicza 112, 90-363 Lodz, Poland; ‡ Department of Structural Chemistry, Centre of Molecular and Macromolecular Studies, Polish Academy of Sciences, Sienkiewicza 112, 90-363 Lodz, Poland

## Abstract

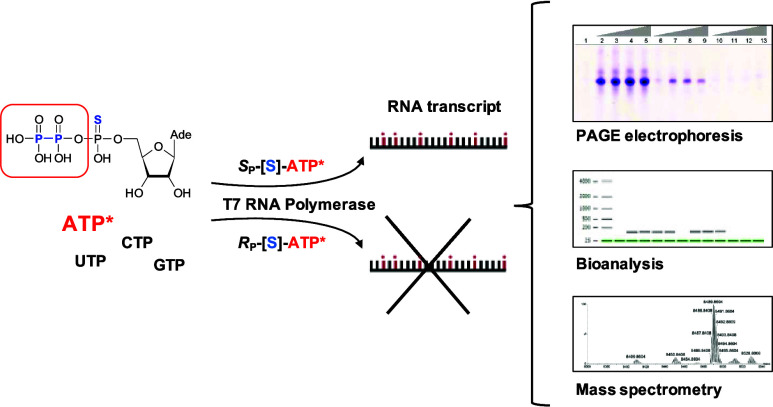

Modified nucleotides are commonly used in molecular biology as substrates or inhibitors for several enzymes but also as tools for the synthesis of modified DNA and RNA fragments. Introduction of modification into RNA, such as phosphorothioate (PS), has been demonstrated to provide higher stability, more effective transport, and enhanced activity of potential therapeutic molecules. Hence, in order to achieve widespread use of RNA molecules in medicine, it is crucial to continuously refine the techniques that enable the effective introduction of modifications into RNA strands. Numerous analogues of nucleotides have been tested for their substrate activity with the T7 RNA polymerase and therefore in the context of their utility for use in *in vitro* transcription. In the present studies, the substrate preferences of the T7 RNA polymerase toward β,γ-hypophospho-modified ATP derivatives for the synthesis of unmodified RNA and phosphorothioate RNA (PS) are presented. The performed studies revealed the stereoselectivity of this enzyme for α-thio-β,γ-hypo-ATP derivatives, similar to that for α-thio-ATP. Additionally, it is demonstrated herein that hypodiphosphoric acid may inhibit *in vitro* transcription catalyzed by T7 RNA polymerase.

## 1 Introduction

Modified nucleosides and nucleotides make up a class of compounds with a wide range of therapeutical applications and utilities in molecular biology. These natural counterparts are known as signaling molecules, agonists of receptors, substrates and cofactors of enzymes, as well as the building blocks for RNA synthesis.^1,2^ Through the multifunctional nature of nucleosides and nucleotides, their analogues are commonly designed and synthesized for the regulation of signaling pathways acting as substrates, inhibitors, cofactors of enzymes, or agonists or antagonists of receptors.^3^ One of the most important applications of modified nucleotides is also their use as building blocks for the synthesis of DNA and RNA.^4,5^ This role of modified nucleotides has recently become particularly important, especially with the widespread utilization of mRNA vaccines to combat the spread of SARS-CoV-2.^6−8^ Nowadays, mRNA-based drugs are considered to open a new era of therapeutic approaches.^9^ Introduction of modifications into RNA has been commonly used for years to provide higher stability and more effective transport and to enhance the activity of such modified molecules.^5,10^

A common way to introduce modifications into the RNA strand is *in vitro* RNA transcription. Several bacteriophage enzymes such as T7, T3, and Sp6 RNA polymerases have been shown to catalyze the *in vitro* synthesis of modified RNA molecules.^5,11^ The T7 RNA polymerase, as one of the most common enzymes involved in RNA production, is particularly interesting due to its wide substrate tolerance.

Certain base-modified nucleotides have been reported to be substrates for T7 RNA polymerase, such as 5-substituted pyrimidine 7-substituted 7-deazapurine nucleoside triphosphates,^12^ 7-ethynyl-8-aza-7-deazaadenosine triphosphate (7-EAATP),^13^ as well as 8-azidoATP,^14^ 2-selenouridine triphosphate,^15^ UTP derivatives modified at the 5-position through an amide linkage,^16^ and others. Nucleotides modified in the sugar part, like 2′-fluoro- and 2′-amino-2′-deoxynucleoside 5′-triphosphates,^17^ 2′-deoxy-2′-α-C-branched nucleoside 5′-triphosphate, such as 5′-triphosphates of 2′-hydroxymethyluridine (2′-homouridine),^18^ or nucleotides containing the 2′-*O*-carbamoyl group^19^ can also be used as substrates for *in vitro* transcription with this enzyme. The mutants of T7 RNA polymerase can even recognize 5′-*O*-triphosphates of 2′-*O*-methyl-, as well as 2′-*O*-fluoro-ribonucleosides, 2′-azido-2′-deoxyribonucleosides, and other 2′-modified nucleotides.^20−22^

The T7 RNA polymerase is also used to introduce modifications into the internucleoside linkages,^10^ such as phosphorothioate (PS),^23^ borano,^24^ or phosphoroselenoate^25,26^ modification. Although phosphorothioate is commonly introduced into synthetic RNA and DNA to enhance their biological activity, interestingly, this modification has also been detected in naturally occurring nucleic acids,^27−29^ which makes it particularly interesting.

The PS modification has been shown to enhance stability^30^ and improve translation of such modified mRNA, allowing more efficient synthesis of proteins.^31^ The T7 RNA polymerase is known to recognize 5′-triphosphates of α-thio-nucleosides in a stereocontrolled manner.^23,32,33^ Only the *S*_P_ diastereoisomer of adenosine 5′-O-(1-thiotriphosphate) was shown to be incorporated into the RNA strand, whereas the *R*_P_ diastereoisomer was neither a substrate nor a competitive inhibitor of the enzyme.^23^ A similar effect was observed in the synthesis of phosphoroselenoate, where also only the *S*_P_ isomer of adenosine 5′-(α-*P*-seleno)triphosphate was used as a substrate by T7 RNA polymerase.^25^ P-stereoselectivity was also demonstrated toward diastereomers of 5′-(α-*P*-borano)triphosphates.^33^

Investigation of compounds that can interact with T7 RNA polymerase is important, not only in the context of their substrate activity and potential utility for incorporation of modifications into RNA strands via *in vitro* transcription but also in terms of their potential inhibitory activity. Based on some structural similarities in RNA polymerases, the data obtained for T7 RNA polymerase may also be useful for the rational design of antiviral agents.^34^

## 2 Results and Discussion

### 2.1 Preparation of the Experimental Model for *In Vitro* Transcription Studies

**Figure 1:**
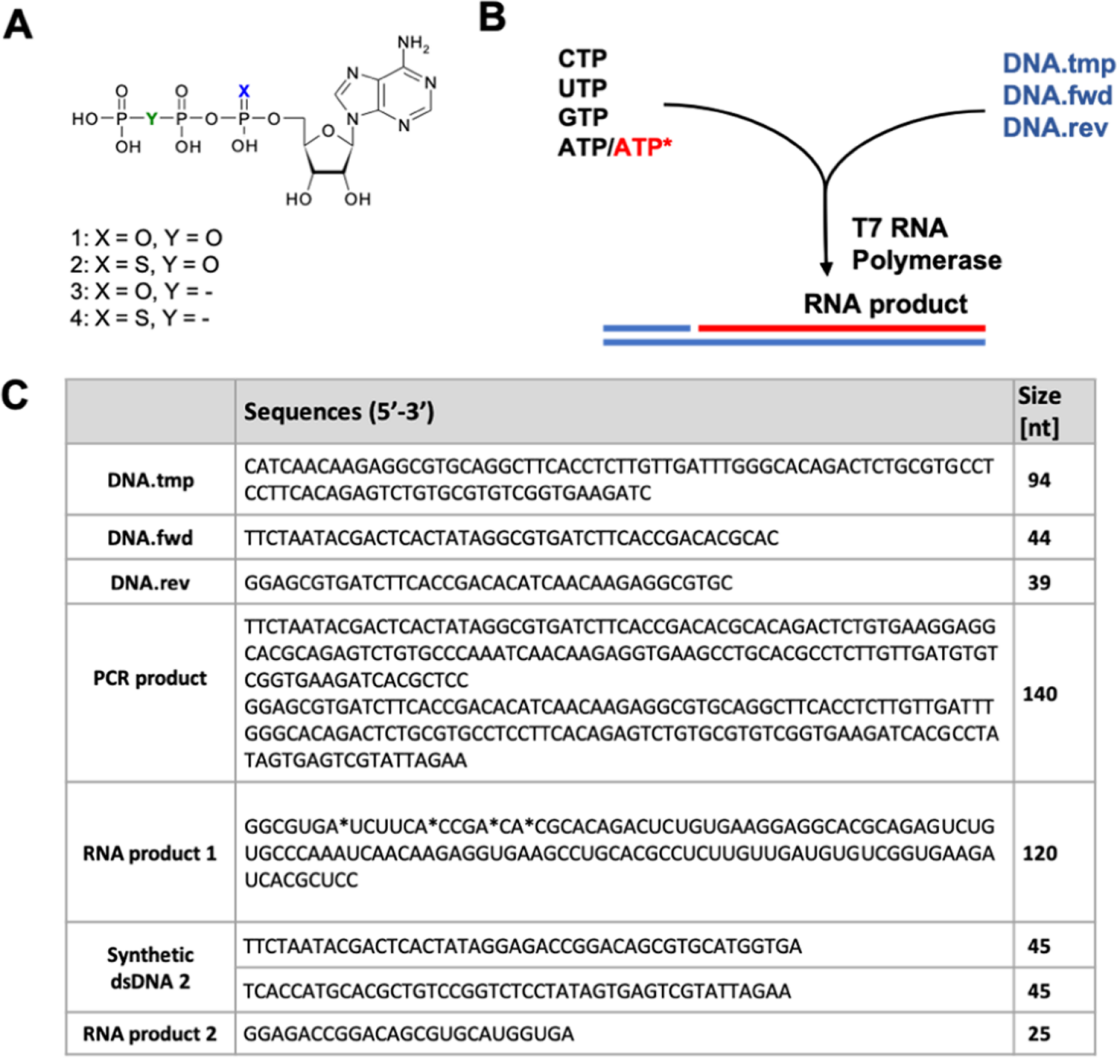
General outline; the experimental model for *in vitro* transcription studies using ATP analogues. (A) Series of ATP analogues used in current studies. (B) Schematic representation of the transcription *in vitro* experimental model. (C) Sequences of template, primers, and RNA products.

The aim of this study was to verify the utility of α-thio-β,γ-hypo-ATP (**4**) derivatives for the synthesis of phosphorothioate (PS)-modified RNA molecules via the *in vitro* transcription method using T7 RNA polymerase. For the comparison of sulfur incorporation efficiency and for enzyme preferences toward individual diastereomers, both β,γ-hypo-ATP (**3**) and α-thio-ATP (**2**) derivatives were also synthesized and analyzed as substrates for T7 RNA polymerase (Figure 1). All analogues were obtained via the oxathiaphospholane method and separated into individual diastereomers, as described previously^1,35^ using a two-step purification protocol. After initial purification on Sephadex resin, α-thio–modified ATP analogues were separated into individual P-diastereomers, i.e., fast and slow according to their relative chromatographic mobility, using revered-phase high-performance liquid chromatography (RP-HPLC; for details, see M&M section). After the separation, the purity of the products was verified by analytical HPLC and confirmed by NMR spectroscopy and high-resolution mass spectrometry (see the Supporting Information). The absolute configuration at the P-stereogenic center in the synthesized α-thio-modified ATP analogues was assigned based on ^1^H NMR spectroscopy and the relative mobility during RP-HPLC analysis. The first indication of a possible stereoconfiguration of compounds was chromatographic mobility. Previous data had indicated that diastereomers with an *S*_P_ configuration have often a shorter retention time during RP-HPLC separation than their *R*_P_ counterparts.^36,37^ The NMR analysis was based on previous studies, where it was demonstrated that in the case of β,γ-modified α-thio-ATP derivatives, the H8 signal should be more shielded in the *S*_P_ isomer than in the isomer *R*_P_ as a result of the influence of the negatively charged α phosphorus moiety.^38,39^ Our observations also indicated that fast-eluting compounds **2** and **4** have an *S*_P_ configuration, whereas slow-eluting ones have an *R*_P_ configuration (8.48 vs 8.42 and 8.56 vs 8.49 for isomers fast and slow of compounds **2** and **4**, respectively). Besides the NMR and RP-HPLC migration rate results, the stereoconfiguration of α-thio-ATP (**2**) derivatives was also confirmed by coinjection with commercially available *S*_P_ and *R*_P_ diastereomers.

To test the substrate activity of the modified ATP analogues the *in vitro* transcription experiments were performed following a previously described model.^40^ In the first step, in order to obtain the double-stranded, dsDNA matrix fragment, the DNA template and appropriate primers were used for DNA amplification. Then, based on the obtained dsDNA fragment, the complementary RNA strand was synthesized through the *in vitro* RNA transcription catalyzed by T7 RNA polymerase (Figure 1). The products of the reaction were analyzed via polyacrylamide gel electrophoresis (PAGE), ultraperformance liquid chromatography coupled with mass spectrometry (UPLC-ESI(−)-MS), and a microfluidic analyzer.

### 2.2 *In Vitro* Transcription with Hypo-Modified ATP Analogues as Substrates for T7 RNA Polymerase

**Figure 2:**
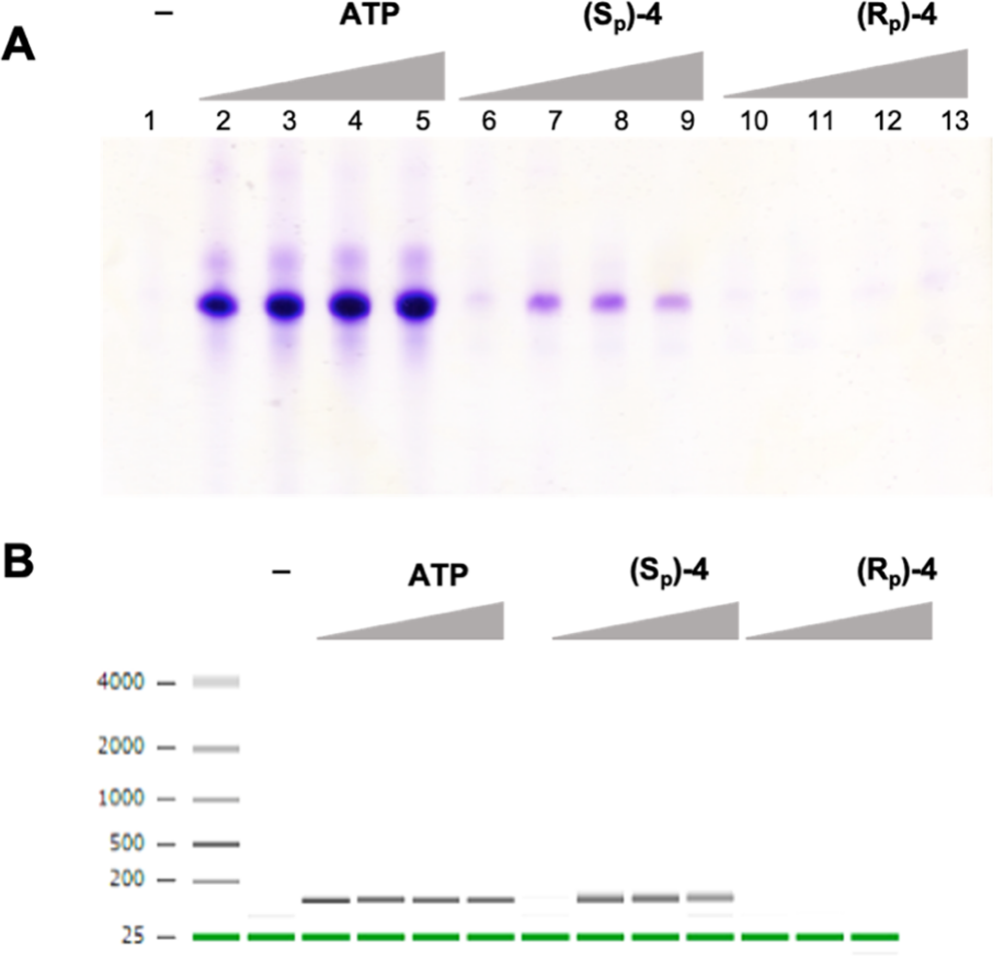
*In vitro* transcription using diastereomerically pure α-thio-β,γ-hypo-ATP (4) analogues as substrates for T7 RNA polymerase. ATP and its analogues were used at concentrations of 0.1, 0.5, 1, and 2 mM. (A) PAGE analysis of the transcription reaction mixture. (B) Determination of the reaction products by microfluidics bioanalysis.

The diastereomerically pure α-thio-β,γ-hypo-ATP (*S*_P_-**4** and *R*_P_-**4**) analogues were analyzed for their substrate activity with the T7 RNA polymerase. The 120 nt length RNA products were synthesized based on the 140nt dsDNA matrix in the *in vitro* transcription reaction. The RNA molecules were separated and detected by polyacrylamide gel electrophoresis and using the Agilent 2100 Bioanalyzer (Agilent). Both PAGE and bioanalysis results indicated that only the *S*_P_ diastereomer of α-thio-β,γ-hypo-ATP (*S*_P_-**4**) may be a substrate for T7 RNA polymerase, whereas the use of its *R*_P_ counterpart did not lead to obtaining the RNA product (Figure 2).

The stereoselectivity of T7 RNA polymerase for *S*_P_ diastereomers of α-thio-ATP and other α-modified nucleoside triphosphates has been reported previously.^23,32,33^ The results obtained herein indicated that the T7 RNA polymerase exhibits stereoselective activity not only for α-thio- but also for the α-thio-β,γ-hypo-modified substrates.

**Figure 3:**
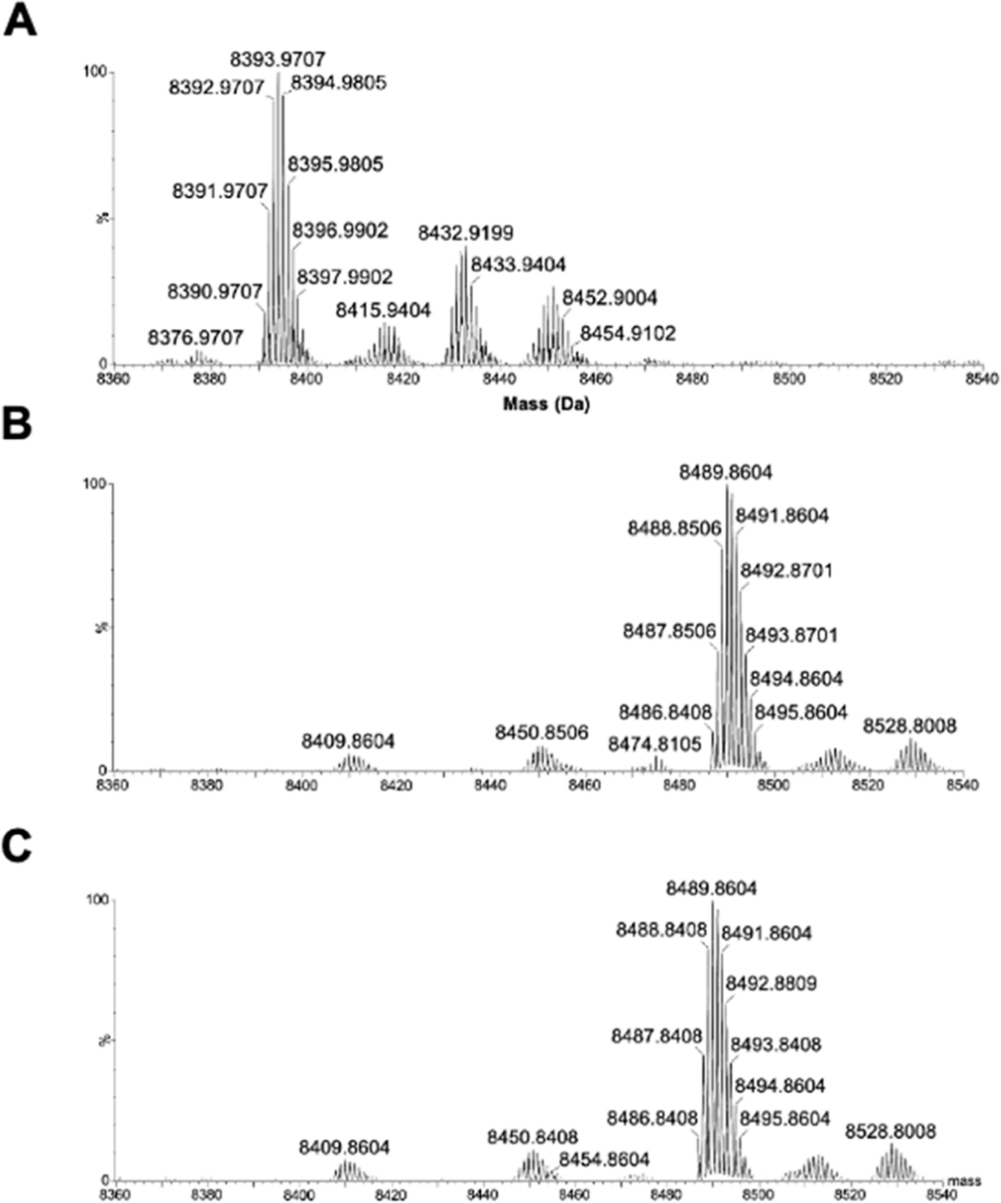
Mass spectrometry analysis of the RNA strand synthesized using (A) ATP (1), (B) (*S*_P_)-α-thio-ATP (2), and (C) (*S*_P_)-α-thio-β,γ-hypo-ATP (4) as substrates for T7 RNA polymerase. All results were obtained after deconvolution of the raw ESI mass spectra using the MaxEnt1 algorithm to a zero-charge state mass.

In order to confirm whether the *S*_P_ diastereomer of α-thio-β,γ-hypo-ATP (*S*_P_**-4**) actually acts as a substrate for T7 RNA polymerase, and whether the RNA product of synthesis contains the phosphothioate modification, the product of *in vitro* transcription was purified and analyzed using mass spectrometry. For the comparison with (*S*_P_)-α-thio-β,γ-hypo-ATP, a control reaction was also performed using (*S*_P_)-α-thio-ATP (*S*_P_**-2**) and ATP (**1**) as known substrates for this enzyme, where the expected products were phosphorothioate RNA (PS-RNA) for *S*_P_**-2** and an unmodified RNA strand for **1**, respectively. Mass spectrometry analysis has proven the introduction of phosphorothioate modification into the newly synthesized RNA strand during the *in vitro* transcription process when the (*S*_P_)-α-thio-β,γ-hypo-ATP (*S*_P_**-4**) molecule was used as a substrate for the *in vitro* synthesis catalyzed by T7 RNA polymerase (Figure 3).

The results obtained for α-thio-β,γ-hypo-ATP (**4**) are in accordance with numerous literature data indicating selectivity toward the *S*_P_ diastereomer of α-thio-modified ATP derivatives.^23,32,33^ Based on these results, we propose the analysis of T7 RNA polymerase activity as an indirect method for the determination of the stereochemical configuration of α-thio-modified nucleotides containing additional modifications such as β,γ-substitution.

**Figure 4:**
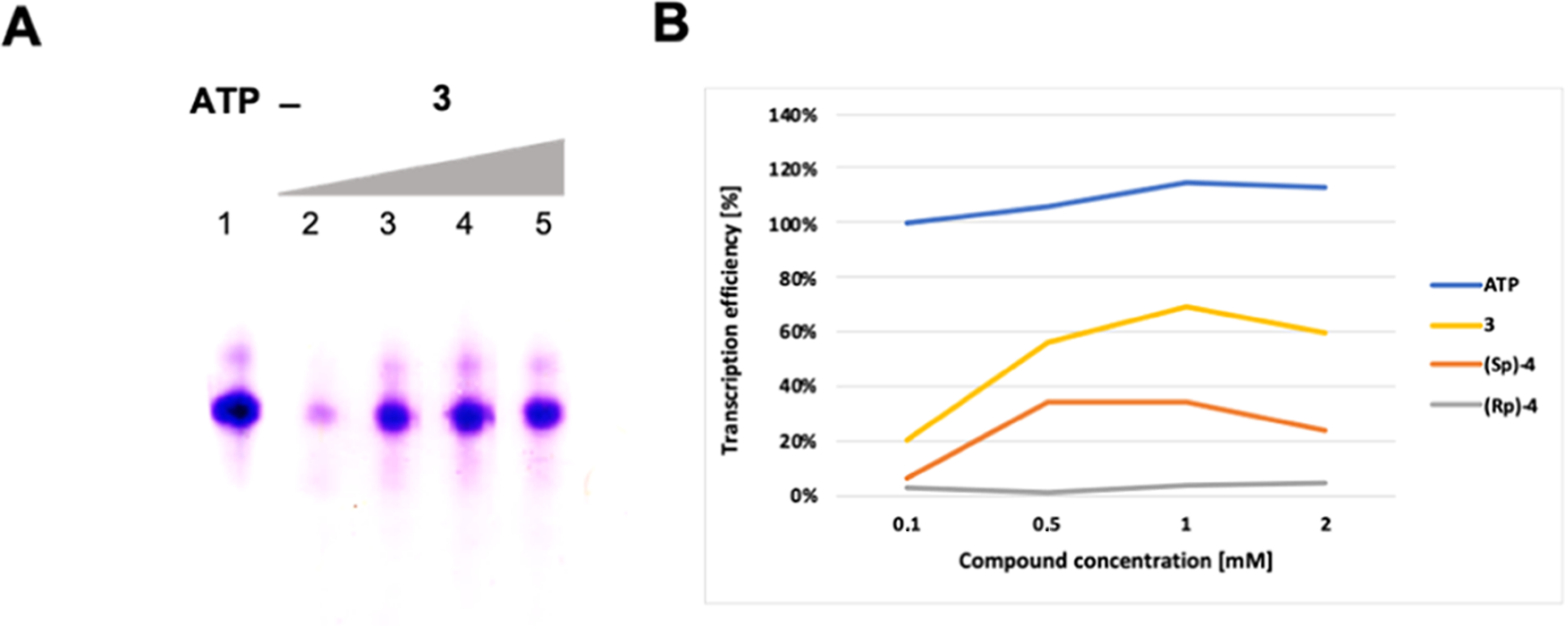
I*n vitro* transcription efficacy using β,γ-hypo-modified ATP derivatives. (A) PAGE analysis of the reaction products when β,γ-hypo-ATP (3) was used as a substrate for T7 RNA polymerase. Lines 2–5 represent reactions at different concentration points: 0.1, 0.5, 1, and 2 mM, respectively. (B) Densitometric analysis of the *in vitro* transcription efficacy with ATP or β,γ-hypo-modified ATP derivatives.

Besides T7 RNA polymerase stereoselectivity, the differences in the reaction yield when using β,γ-hypophospho-modified ATP analogues compared to natural substrates were detected. The decrease of *in vitro* efficacy was observed not only when using (*S*_P_)-α-thio-β,γ-hypo-ATP (4), where the PS-RNA was produced, but also for β,γ-hypo-ATP (3), where the expected product was unmodified RNA (Figure 4). This observation suggests potential differences in the substrate preferences of T7 RNA polymerase toward β,γ-hypo-modified ATP derivatives compared to unmodified counterparts or the inhibitory activity of hypodiphosphate produced during RNA synthesis from these derivatives.

### 2.3 Substrate Preferences of T7 RNA Polymerase toward α-Thio-Modified ATP Derivatives in the Presence of ATP

In order to compare substrate preferences of T7 RNA polymerase toward α-thio-modified **2** and **4** derivatives, the *in vitro* transcription reaction was performed in the presence of unmodified ATP and *S*_P_ diastereomers of α-thio-β,γ-hypo-ATP (**4**) or α-thio-ATP (**2**), respectively.

**Figure 5:**
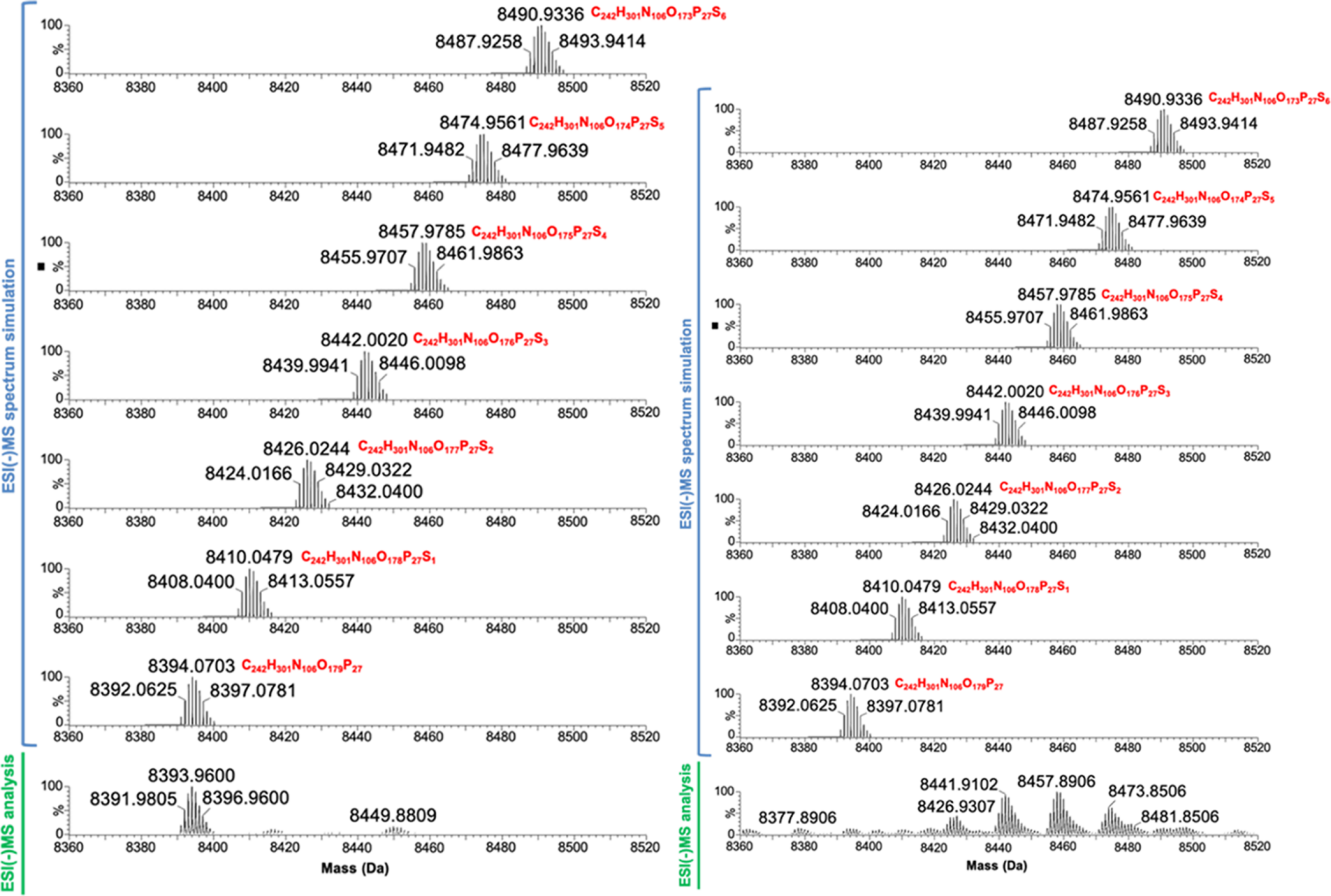
Mass spectrometry analysis of T7 RNA polymerase substrate preferences toward ATP and α-thio-modified ATP analogues. The detection of PS-modified RNA as a product of *in vitro* transcription conducted in the presence of ATP and α-thio-modified ATP analogues: ATP with *S*_P_-**4** (upper panel) or in the presence of *S*_P_-**2** with ATP (lower panel). From the top, simulated mass spectra of unmodified and PS-modified products containing from 1 to 6 phosphorothioate modifications. The lowest graphs in each part (in the green part) present experimental results of the MS analysis of transcription reaction products. The results were obtained after deconvolution of the raw ESI mass spectra using the MaxEnt1 algorithm to a zero-charge state mass.

The mass spectrometry analysis indicated that although *S*_P_-**4** could serve as a substrate for the synthesis of PS-modified RNA (Figures 2 and 3), if unmodified ATP was present in the mixture, the main product of *in vitro* transcription catalyzed by T7 RNA polymerase was the unmodified RNA. The RNA containing the phosphorothioate modification was present; however, the fully modified RNA was not produced or was produced at yields below the detection threshold when the mix *S*_P_-α-thio-β,γ,-hypo-ATP/ATP was used for the reaction (Figure 5 left). Interestingly, using *S*_P_-α-thio-ATP under the same conditions resulted in obtaining expected PS-modified products (Figure 5 right). This disproportion suggested the different affinity of α-thio-ATP (**2**) and α-thio-β,γ-hypo-ATP (**4**) toward T7 RNA polymerase, the different specificity of interaction with the substrates, or the inhibitory activity of one of the products formed during the reaction. Mass spectra of the [M-9H^+^]^9–^ ion of the reaction products in the presence of the mixture **1**/*S*_P_-**2** and **1**/*S*_P_-4, as well as simulated mass spectra of unmodified and PS-modified products, are given in the Supporting Information (Figure S22).

### 2.4 Efficacy of RNA Synthesis by T7 RNA Polymerase in the Presence of α-Thio-β,γ-hypophospho-Modified ATP Derivatives

**Figure 6:**
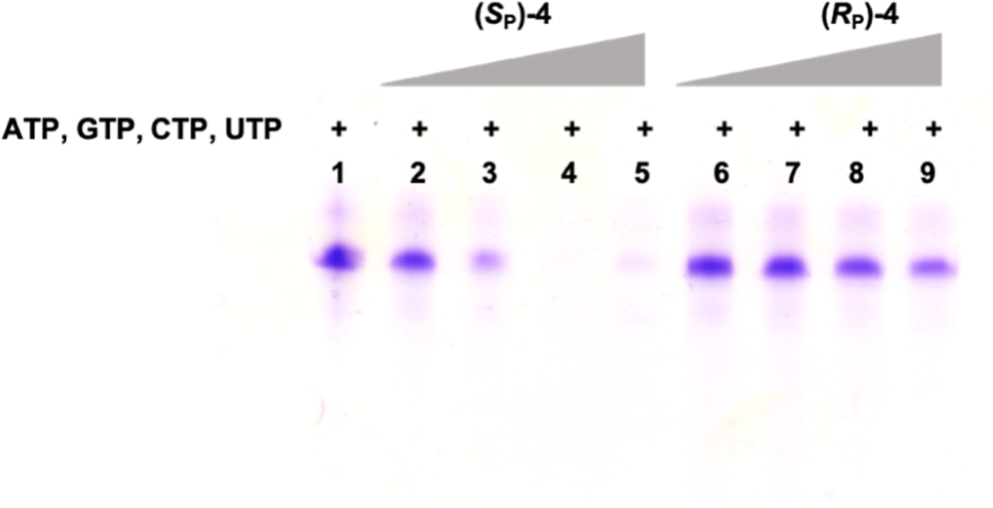
PAGE analysis of the *in vitro* transcription efficacy in the presence of *S*_P_ and *R*_P_ diastereomers of α-thio-β,γ-hypo-ATP (4) used at concentrations of 0.1, 0.5, 1, and 2 mM.

In order to verify whether the presence of α-thio-β,γ-hypo-ATP (**4**) derivatives may affect the T7 RNA polymerase activity, an *in vitro* transcription was performed using all canonical nucleoside triphosphates (GTP, CTP, UTP, and ATP) and increasing quantities of *S*_P_ or *R*_P_ diastereomers of α-thio-β,γ-hypo-ATP (**4**), respectively. Tested ATP analogues were used at concentrations 0.1, 0.5, 1, and 2 mM, with 0.5 mM concentration of the remaining nucleotide triphosphates (NTPs), which leads to the following ratio of ATP analogue to ATP: 0.2:1, 1:1, 2:1, and 4:1. The obtained results clearly showed a reduced yield of the RNA product when using *S*_P_-α-thio-β,γ-hypo-ATP (*S*_P_-**4**), which was demonstrated to be a substrate for T7 RNA polymerase compared to its unreacted *R*_P_ counterpart (*R*_P_-**4**) (Figure 6).

Additionally, the PAGE analysis of the *in vitro* transcription demonstrated that an increased amount of *S*_P_-α-thio-β,γ-hypo-ATP (*S*_P_**-4**) derivatives resulted in no detection of the reaction product despite the presence of necessary nucleotides (GTP, CTP, UTP, and ATP), which was not observed for the *R*_P_ diastereomer (*R*_P_**-4**). One of the possible explanations for this phenomenon could be the partial inhibitory activity of the *S*_P_ derivative. Another possibility is that at high concentrations of the *S*_P_-**4** diastereomer, when a larger amount of substrate is used, leading to an increased amount of the reaction product, the activity of the enzyme may be inhibited. This corroborates with the fact that such a strong inhibition was not observed for the *R*_P_ diastereomer of **4**, which was not a substrate and therefore did not lead to the formation of the hypodiphosphate.

### 2.5 *In Vitro* Transcription in the Presence of Pyro- and Hypodiphosphoric Acid

**Figure 7:**
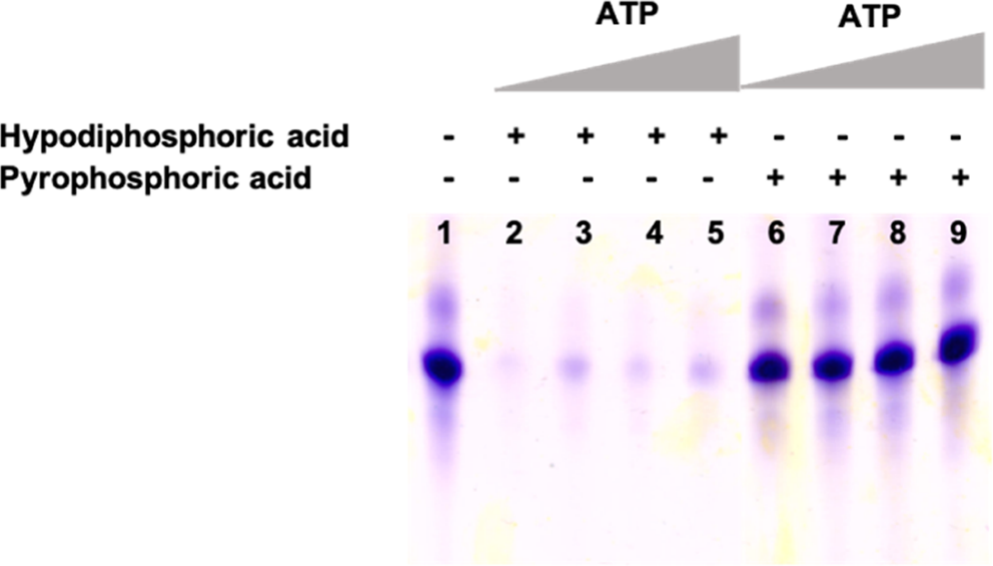
PAGE analysis of the *in vitro* transcription efficiency in the presence of hypodiphosphoric and pyrophosphoric acids with an increasing amount of ATP, lines 2–5 and 6–9: 0.1, 0.5, 1, and 2 mM ATP, respectively.

One of the most probable explanations for the decreased efficacy of the *in vitro* transcription reaction in the presence of *S*_P_-**4** seemed to be the inhibitory activity of hypodiphosphoric acid, which is the product of the T7 RNA polymerase reaction, when β,γ-hypo-modified derivatives were used as effective substrates. In order to verify this hypothesis, *in vitro* transcription was performed in the presence of hypodiphosphoric acid and pyrophosphoric acid using unmodified NTP substrates, separately (Figure 7).

Indeed, the electrophoretic analysis of the *in vitro* transcription mixture showed the inhibition of the reaction catalyzed by the T7 RNA polymerase in the presence of hypodiphosphoric acid, whereas at the same concentration, the reaction was not affected by pyrophosphoric acid (Figure 7). This result clearly confirmed the inhibitory activity of hypodiphosphoric acid for T7 RNA polymerase. Whereas a slight decrease of the *in vitro* transcription activity with a higher concentration of pyrophosphoric acid has been previously reported, and this was observed in our studies (Figure S23), the inhibitory activity of hypodiphosphoric acid toward T7 RNA polymerase has never been demonstrated. Hypodiphosphoric acid has been considered to interact with placental alkaline phosphatase (PLAP)^41^ and tissue-nonspecific alkaline phosphatase (TNAP)^42^ and was demonstrated to be a substrate of pyrophosphorolysis catalyzed by HIV-1 reverse transcriptase;^43^ however, to date, little is known about the other biological properties of this molecule.^44^ Thus, the obtained results make a significant contribution toward the knowledge about this molecule and the potential activity of its derivatives.

## 3 Conclusions

We have demonstrated here, for the first time, the substrate preferences of T7 RNA polymerase for selected β,γ-hypo-modified ATP analogues, thus indicating their potential utility in the *in vitro* synthesis of both unmodified RNA and phosphorothioate RNA. Despite the unquestionable advantages provided by phosphorothioate modification, the determination of the absolute stereoconfiguration of such modified nucleotides meets some difficulties. The T7 RNA polymerase is one of the enzymes known to stereoselectively recognize the *S*_P_ diastereomer of α-thio-ATP. Herein, we have indicated that stereoselectivity of the enzyme may be applicable to α-thio-modified nucleotide derivatives with β,γ-modification, which makes it a potential tool for assignment of absolute configuration of not only α-thio- but also α-thio-β,γ-modified nucleotides.

Additionally, for the first time, hypophosphoric acid was demonstrated to decrease the efficiency of the *in vitro* transcription catalyzed by T7 RNA polymerase. Our observation opened a new way for studying the mechanistic properties of T7 RNA polymerase action, its substrate activity toward various β,γ-modified nucleotides, and possible inhibition by bisphosphonates formed as reaction products. This type of mechanistic study may lead to the understanding of efficient and effective synthesis of modified RNAs and is particularly valuable in terms of optimizing the production and introducing further RNA-based therapeutics to the market. Moreover, the model in which the inhibitory effect on T7 RNA polymerase is caused by the product of its action is interesting in the context of potential inhibition of viral RNA polymerases and the rational design of antiviral drugs.

## 4 Materials and Methods

### 4.1 Synthesis of ATP Analogues

The adenosine 5′-*O*-(1-thiotriphosphate) analogues, presented in this paper, were synthesized via the oxathiaphospholane method^45,46^ according to the procedure described previously.^1^ The protected adenosine 5′-*O*-(2-thio-1,3,2-oxathiaphospholane) in the presence of DBU as a base catalyst was reacted with pyrophosphate or hypophosphate for compounds **2** and **4**, respectively. The ring-opening reaction followed by the spontaneous elimination of ethylene sulfide led to the desired analogues. The reactions were performed at room temperature with the exclusion of moisture. After the deprotection step, with 25% aqueous ammonia, the compounds were purified by ion-exchange chromatography (DEAE-Sephadex) using triethylammonium bicarbonate (TEAB) as an eluent. The β,γ-hypo-ATP (**3**) was obtained from the starting α-thio-β,γ-hypo-ATP (**4**) (as a diastereomeric mixture) using iodoxybenzene according to a previously published protocol.^1^ The obtained compounds were additionally purified and separated into the individual P-diastereoisomers using high-performance liquid chromatography (RP-HPLC) with linear gradient 0–30% MeCN supplemented with 0.1 mol/L triethylammonium acetate buffer (TEAAc) (pH 7.5). The final quality of the compounds was achieved by analytical RP-HPLC analysis.

### 4.2 Preparation of dsDNA Template

The DNA template, used for transcription studies, was prepared by amplification using a polymerase chain reaction (PCR). For PCR, the DNA template strand (DNA.tmp) with the DNA forward strand (DNA.fwd) and the DNA reverse strand (DNA.rev), with the sequence presented in Figure 1, was used. Each PCR was carried out in 100 μL volume, and the reaction mix consisted of 0.004 μM DNA template, 1 μM primer forward, 1 μM primer reverse, and 1× PCR master mix (PCR Plus mixture solution from A&A Biotechnology). Both primer strands and the DNA template strand were purchased from Genomed. Concentrations of all oligonucleotides were estimated using a NanoDrop spectrophotometer (ThermoScientific).

### 4.3 *In Vitro* Transcription

The T7 RNA *in vitro* transcription was performed based on the procedures described previously.^40,47^ The transcription reaction was carried out in a 50 μL final volume by combining equimolar amounts of three nucleoside triphosphates, 0.5 mM GTP, UTP, and CTP, and selected ATP analogues. For the positive control, 0.5 mM GTP, UTP, CTP, and ATP were used. The negative control was prepared without ATP or ATP analogues. For the assessment of reaction efficiency, the ATP or ATP derivative was used at concentrations of 0.1, 0.5, 1, and 2 mM. The *in vitro* transcription was performed in the presence of 25 mM MgCl_2_, 40 mM Tris-HCl, 10 mM dithiothreitol, and 2 mM spermidine, at pH = 7.9 with 1 U/μL T7 RNA polymerase (Lucigen). The reaction was carried out at 37 °C for 4 h and stopped by addition of 25 μL of a 7 M urea–40 mM EDTA buffer and heating to 90 °C for 3 min.

### 4.4 T7 RNA Polymerase Efficiency in the Presence of Tested Compounds

The enzymatic mix was prepared in a 50 μL final volume by combining equimolar amounts of four nucleoside triphosphates, 0.5 mM GTP, UTP, CTP, and ATP, and a variable concentration of tested compounds. In cases of hypo- and pyrophosphate impact studies, the reaction was performed in the presence of 0.5 mM hypodiphosphoric acid or pyrophosphoric acid, respectively, with an increasing concentration of ATP. Selected ATP analogues or ATP was titrated to the final concentrations of 0.1, 0.5, 1, and 2 mM. Transcription was carried out in the presence of 25 mM MgCl_2_, 40 mM Tris-HCl, 10 mM dithiothreitol, and 2 mM spermidine, at pH = 7.9 and 1 U/μL T7 RNA polymerase (Lucigen). The transcription reaction was carried out at 37 °C for 4 h and stopped by the addition of 25 μL of a 7 M urea–40 mM EDTA buffer and heating to 90 °C for 3 min.

### 4.5 Polyacrylamide Gel Electrophoresis Analysis

The reaction mixtures were analyzed with a 10% denaturing polyacrylamide gel. Electrophoresis was performed using 10% acrylamide:bis-acrylamide gel (19:1) in 7 M urea, 50 mM TRIS, 50 mM boric acid, and 1 mM EDTA. The electrophoresis was run for 3 h at 300 V at room temperature. The RNA products were visualized using a Stains-all solution (Stains-all 50 μg/mL (Sigma-Aldrich), 10% formamide, 25% isopropanol, 50 mM TRIS, 50 mM boric acid). After overnight staining, the PAGE gels were washed three times with Milli-Q water. Gel images were analyzed densitometrically by using ImageJ software. Results were normalized to an internal positive control with ATP, which was taken as 100%. The remaining results were estimated in relation to this value.

### 4.6 Bioanalysis

Bioanalysis of T7 RNA polymerase reaction products was performed using the Agilent 2100 Bioanalyzer (Agilent). For analysis, “RNA nano chip” (Agilent) and the “Eukaryote total RNA Nano II” bioanalysis program were used. Analysis was performed using 1 μL from each transcription sample. Procedures were performed in accordance with the manufacturer’s recommendations.

### 4.7 Preparation of Samples for Mass Spectrometry Analysis

For the mass spectrometry analysis, the *in vitro t*ranscription reaction was scaled up 4-fold from the 50 to 200 μL final volume. Master mix was prepared by mixing 0.5 mM UTP, GTP, and CTP with 19 mM MgCl_2_ and T7 polymerase buffer, DNA template, and T7 polymerase. Each sample contains 25 mM Mg^2+^ from the buffer and MgCl_2_ solution. RNA-ase free water was added first to 500 μL tubes, and then, appropriate amounts of ATP or ATP analogues were added and supplemented with the master mix. Short, synthetic 10nt dsDNA was used as a template.

Transcription was performed for 4 h at 37 °C in 200 μL volume. After transcription, samples were mixed with 100 μL of urea/EDTA and incubated at 90 °C for 3 min. Then, samples were applied on the 10% PAGE gel and 7 M urea (20 mL of 20% acrylamide:bis-acrylamide 19:1, 7 M urea, 16 mL of 7 M urea, 4 mL of 10x TBE). The gel was run at 300 V for 2 h. Then, RNA samples were extracted from the gel.

For this purpose, RNA bands were cut out from the gel and placed in Eppendorf tubes with 600 μL of elution buffer. Gel samples were incubated overnight at 8 °C with constant mixing at 350 rpm. A solution of the eluent from overnight incubation was separated from the acrylamide gel pieces and gently mixed with 100% ice-cold ethanol and incubated in a freezer (−20 °C) for a minimum of 2 h. After that, the samples were washed. For this purpose, tubes were centrifuged for 20 min, 12,000 rpm at 4 °C, and the liquid was removed. Then, 70% ethanol was added to the pellet and samples were centrifuged again for 10 min, 12,000 rpm at 4 °C. After removing of the supernatant, purified RNA was dried in the Speedvac and resuspended in RNase and DNase free water. The concentration of RNA was measured using Nanodrop. Finally, samples were dried using a Speedvac centrifuge, and mass spectrometry analysis was performed.

### 4.8 Mass Spectrometry

All samples were analyzed using an ACQUITY UPLC I-Class chromatography system equipped with a photodiode array detector with a binary solvent manager (Waters Corp., Milford, MA) coupled with a SYNAPT G2-S*i* mass spectrometer equipped with an electrospray source and a quadrupole time-of-flight mass analyzer (Waters Corp., Milford, MA). The ACQUITY UPLC Oligonucleotides BEH C18 column (50 mm × 2.1 mm, 1.7 μm) maintained at 60 °C temperature was used for the chromatographic separation of the analyte. A gradient program was employed with the mobile phase combining solvent A (15 mM triethylamine, 400 mM hexafluoroisopropanol in water) and solvent B (50% methanol, 50% solvent A, v/v) as follows: 25% B (0–0.2 min), 25–70% B (0.5–10.0 min), 70–70% B (10.0–13.0 min), 70–25% B (13.0–13.2 min), and 25–25% B (13.2–15 min). The flow rate was 0.2 mL/min, and the injection volume was 5 μL.

For mass spectrometric detection, the electrospray source was operated in the negative resolution mode. The optimized source parameters were as follows: capillary voltage 2.7 kV, cone voltage 40 V, desolvation gas flow 600 L/h with the temperature 400 °C, nebulizer gas pressure 6.5 bar, and source temperature 120 °C. Mass spectra were recorded over an *m*/*z* range of 500–2000. Mass spectrometer conditions were optimized by the direct infusion of the standard solution. The system was controlled by using MassLynx software (Version 4.1). The raw ESI mass spectra were deconvoluted by using the MaxEnt1 algorithm to a zero-charge state mass.

## Supporting Information Available

Figures S1–S21, ^1^H NMR and ^31^P NMR spectra, mass spectrometry and HPLC profiles of all synthesized compounds; Figure S22, mass spectrometry analysis of the [M-9H^+^]^9–^ ion of the reaction products of the *in vitro* transcription conducted in the presence of ATP and α-thio-modified ATP analogues; Figure S23, PAGE analysis of the *in vitro* transcription efficiency in the presence of an increasing amount of pyrophosphoric acid; and Table S1, calculated and experimentally confirmed monoisotopic mass of RNA strands synthesized by the T7 RNA polymerase in the presence of ATP and α-thio-modified ATP analogues (PDF)

## Abbreviations

PS: phosphorothioate
PAGE: polyacrylamide gel electrophoresis
UPLC: ultraperformance liquid chromatography
MS: mass spectrometry
PLAP: placental alkaline phosphatase
TNAP: tissue-nonspecific alkaline phosphatase

## References

[ref1] Pawlowska R.; Korczynski D.; Nawrot B.; Stec W. J.; Chworos A. The α-thio and/or β-γ-hypophosphate analogs of ATP as cofactors of T4 DNA ligase. Bioorg. Chem. 2016, 67, 110–115. 10.1016/j.bioorg.2016.06.003. 27337226

[ref2] Suwara J.; Radzikowska-Cieciura E.; Chworos A.; Pawlowska R. The ATP-dependent Pathways and Human Diseases. Curr. Med. Chem. 2023, 30, 1232–1255. 10.2174/0929867329666220322104552. 35319356

[ref3] Pawlowska R.; Chworos A. Nucleoside and Nucleotide Analogues as Potential Therapeutics. Curr. Med. Chem. 2023, 30, 1207–1208. 10.2174/092986733011230106124249. 36872461

[ref4] Jedrzejczyk D.; Gendaszewska-Darmach E.; Pawlowska R.; Chworos A. Designing synthetic RNA for delivery by nanoparticles. J. Phys.: Condens. Matter 2017, 29 (12), 123001 10.1088/1361-648X/aa5561. 28004640

[ref5] Graczyk A.; Radzikowska-Cieciura E.; Kaczmarek R.; Pawlowska R.; Chworos A. Modified Nucleotides for Chemical and Enzymatic Synthesis of Therapeutic RNA. Curr. Med. Chem. 2023, 30, 1320–1347. 10.2174/0929867330666221014111403. 36239720

[ref6] Corbett K. S.; Edwards D. K.; Leist S. R.; Abiona O. M.; Boyoglu-Barnum S.; Gillespie R. A.; Himansu; et al. SARS-CoV-2 mRNA vaccine design en- abled by prototype pathogen preparedness. Nature 2020, 586, 567–571. 10.1038/s41586-020-2622-0. 32756549 PMC7581537

[ref7] Polack F. P.; Thomas S. J.; Kitchin N.; Absalon J.; Gurtman; et al. Safety and efficacy of the BNT162b2 mRNA COVID-19 vaccine. N. Engl. J. Med. 2020, 383, 2603–2615. 10.1056/NEJMoa2034577. 33301246 PMC7745181

[ref8] Baden L. R.; El Sahly H. M.; Essink B.; Kotloff K.; Frey; et al. Efficacy and safety of the mRNA-1273 SARS-CoV-2 vaccine. N. Engl. J. Med. 2021, 384 (5), 403–416. 10.1056/NEJMoa2035389. 33378609 PMC7787219

[ref9] Sahin U.; Karikó K.; Türeci Ö. mRNA-based therapeutics — developing a new class of drugs. Nat. Rev. Drug Discovery 2014, 13 (10), 759–780. 10.1038/nrd4278. 25233993

[ref10] Clavé G.; Reverte M.; Vasseur J. J.; Smietana M. Modified internucleoside linkages for nuclease-resistant oligonucleotides. RSC Chem. Biol. 2021, 2, 94–150. 10.1039/D0CB00136H. 34458777 PMC8341215

[ref11] Beckert B.; Masquida B. Synthesis of RNA by in vitro transcription. Methods Mol. Biol. 2011, 703, 29–41. 10.1007/978-1-59745-248-9_3. 21125481

[ref12] Milisavljevič N.; Perlíková P.; Pohl R.; Hocek M. Enzymatic synthesis of base-modified RNA by T7 RNA polymerase. A systematic study and comparison of 5-substituted pyrimidine and 7-substituted 7-deazapurine nucleoside triphosphates as substrates. Org. Biomol. Chem. 2018, 16, 5800–5807. 10.1039/C8OB01498A. 30063056

[ref13] Zheng Y.; Beal P. A. Synthesis and evaluation of an alkyne-modified ATP analog for enzymatic incorporation into RNA. Bioorg. Med. Chem. Lett. 2016, 26, 1799–1802. 10.1016/j.bmcl.2016.02.038. 26927424 PMC4785081

[ref14] Gopalakrishna S.; Gusti V.; Nair S.; Sahar S.; Gaur R. K. Template-dependent incorporation of 8-N3AMP into RNA with bacteriophage T7 RNA polymerase. RNA 2004, 10, 1820–1830. 10.1261/rna.5222504. 15388871 PMC1370669

[ref15] Sun H.; Jiang S.; Caton-Williams J.; Liu H.; Huang Z. 2-Selenouridine triphosphate synthesis and Se-RNA transcription. RNA 2013, 19, 1309–1314. 10.1261/rna.038075.112. 23887148 PMC3753936

[ref16] Vaught J. D.; Dewey T.; Eaton B. E. T7 RNA polymerase transcription with 5-position modified UTP derivatives. J. Am. Chem. Soc. 2004, 126, 11231–11237. 10.1021/ja049009h. 15355104

[ref17] Aurup H.; Williams D. M.; Eckstein F. 2′-Fluoro- and 2′-amino-2′-deoxynucleoside 5′-triphosphates as substrates for T7 RNA polymerase. Biochemistry 1992, 31, 9636–9641. 10.1021/bi00155a016. 1390741

[ref18] Pavey J. B. J.; Lawrence A. J.; O’Neil I. A.; Vortler S.; Cosstick R. Synthesis and transcription studies on 5′-triphosphates derived from 2′-C-branched-uridines: 2′-homouridine-5′-triphosphate is a substrate for T7 RNA polymerase. Org. Biomol. Chem. 2004, 2, 869–875. 10.1039/B314348A. 15007416

[ref19] Masaki Y.; Ito H.; Oda Y.; Yamazaki K.; Tago N.; Ohno K.; Ishii N.; Tsunoda H.; Kanamori T.; Ohkubo A.; Sekine M.; Seio K. Enzymatic synthesis and reverse transcription of RNAs incorporating 2′-O-carbamoyl uridine triphosphate. Chem. Commun. 2016, 52, 12889–12892. 10.1039/C6CC05796A. 27738673

[ref20] Chelliserrykattil J.; Ellington A. D. Evolution of a T7 RNA polymerase variant that transcribes 2′-O-methyl RNA. Nat. Biotechnol. 2004, 22, 1155–1160. 10.1038/nbt1001. 15300257

[ref21] Ibach J.; Dietrich L.; Koopmans K. R. M.; Nöbel N.; Skoupi M.; Brakmann S. Identification of a T7 RNA polymerase variant that permits the enzymatic synthesis of fully 2′O- -methyl-modified RNA. J. Biotechnol. 2013, 167 (3), 287–295. 10.1016/j.jbiotec.2013.07.005. 23871655

[ref22] Meyer A. J.; Garry D. J.; Hall B.; Byrom M. M.; McDonald H. G.; Yang X.; Yin Y. W.; Ellington A. D. Transcription yield of fully 2′-modified RNA can be increased by the addition of thermostabilizing mutations to T7 RNA polymerase mutants. Nucleic Acids Res. 2015, 43, 7480–7488. 10.1093/nar/gkv734. 26209133 PMC4551944

[ref23] Griffiths A. D.; Potter B. V.; Eperon I. C. (). Stereospecificity of nucleases towards phosphorothioate-substituted RNA: stereochemistry of transcription by T7 RNA polymerase. Nucleic Acids Res. 1987, 15, 4145–4162. 10.1093/nar/15.10.4145. 2438652 PMC340838

[ref24] Wan J.; Shaw B. R. Incorporation of ribonucleoside 5′-(α-P-bor- ano)triphosphates into a 20-mer RNA by T7 RNA polymerase. Nucleosides, Nucleotides Nucleic Acids 2005, 24, 943–946. 10.1081/NCN-200059303. 16248068

[ref25] Carrasco N.; Caton-Williams J.; Brandt G.; Wang S.; Huang Z. Efficient enzymatic synthesis of phosphoroselenoate RNA by using adenosine 5′-(alpha-P-seleno)t- riphosphate. Angew. Chem., Int. Ed. 2005, 45, 94–97. 10.1002/anie.200502215. 16304655

[ref26] Lin L.; Caton-Williams J.; Kaur M.; Patino A. M.; Sheng J.; Punetha J.; Huang Z. (). Facile synthesis of nucleoside 5′-(α-P-seleno)-triphosphates and phosphoroselenoate RNA transcription. RNA 2011, 17, 1932–1938. 10.1261/rna.2719311. 21873462 PMC3185924

[ref27] Wu Y.; Tang Y.; Dong X.; Zheng Y. Y.; Haruehanroengra P.; Mao S.; Lin Q.; Sheng J. RNA phosphorothioate modification in prokaryotes and eukaryotes. ACS Chem. Biol. 2020, 15, 1301–1305. 10.1021/acschembio.0c00163. 32275390

[ref28] Zheng Y. Y.; Wu Y.; Begley T. J.; Sheng J. Sulfur modification in natural RNA and therapeutic oligonucleotides. *RSC*. Chem. Biol. 2021, 2, 990–1003. 10.1039/D1CB00038A. PMC8341892 34458821

[ref29] Pawlowska R.; Guga P. Phosphorothioate Nucleic Acids: Artificial Modification Envisaged by Nature. In Handbook of Chemical Biology of Nucleic Acids; Sugimoto N., Ed.; Springer: Singapore, 2023; pp 1–26.

[ref30] Claus S Vörtler L.; Eckstein F. Phosphorothioate modification of RNA for stereochemical and interference analyses. Methods Enzymol. 2000, 317, 74–91. 10829273 10.1016/s0076-6879(00)17007-7

[ref31] Kawaguchi D.; Kodama A.; Abe N.; Takebuchi K.; Hashiya F.; Tomoike F.; Nakamoto K.; Kimura Y.; Shimizu Y.; Abe H. Phosphorothioate Modification of mRNA Accelerates the Rate of Translation Initiation to Provide More Efficient Protein Synthesis. Angew. Chem., Int. Ed. 2020, 59, 17403–17407. 10.1002/anie.202007111. 32627275

[ref32] Lin L.; Caton-Williams J.; Kaur M.; Patino A. M.; Sheng J.; Punetha J.; Huang Z. Facile synthesis of nucleoside 5′-(α-P-seleno)-triphosphates and phosphoroselenoate RNA transcription. RNA 2011, 17, 1932–1938. 10.1261/rna.2719311. 21873462 PMC3185924

[ref33] Strzelecka D.; Smietanski M.; Sikorski P. J.; Warminski M.; Kowalska J.; Jemielity J. Phosphodiester modifications in mRNA poly(A) tail prevent deadenylation without compromising protein expression. RNA 2020, 26, 1815–1837. 10.1261/rna.077099.120. 32820035 PMC7668260

[ref34] Öberg B. Rational design of polymerase inhibitors as antiviral drugs. Antiviral Res. 2006, 71, 90–95. 10.1016/j.antiviral.2006.05.012. 16820225

[ref35] Błaziak D.; Guga P.; Jagiełło A.; et al. Stereoselective formation of a P-P bond in the reaction of 2-alkoxy-2-thio-1,3,2-oxathiaphospholanes with O,O-dialkyl H-phosphonates and H-thiophosphonates. Org. Biomol. Chem. 2010, 8, 5505–5510. 10.1039/c0ob00104j. 20944857

[ref36] Koziołkiewicz M.; Uznański B.; Stec W. J.; Zon G. P-chiral analogues of oligodeoxyribonu- cleotides: synthesis, stereochemistry and enzyme studies. Chem. Scr. 1986, 26, 251–260.

[ref37] Krakowiak A.; Pawłowska R.; Kocoń-Rębowska B.; Dolot R.; Stec W. J. Interactions of cellular histidine triad nucleotide binding protein 1 with nucleosides 5′-O-monophosphorothioate and their derivatives - Implication for desulfuration process in the cell. Biochim. Biophys. Acta 2014, 1840, 3357–3366. 10.1016/j.bbagen.2014.08.016. 25199874

[ref38] Major D. T.; Fischer B. Molecular recognition in purinergic receptors. 1. A comprehensive computational study of the h-P2Y1- receptor. J. Med. Chem. 2004, 47, 4391–4404. 10.1021/jm049772m. 15317452

[ref39] Nadel Y.; Lecka J.; Gilad Y.; Ben-David G.; Förster D.; Reiser G.; Kenigsberg S.; Camden J.; Weisman G. A.; Senderowitz H.; Sévigny J.; Fischer B. Highly potent and selective ectonucleotide pyrophosphatase/phosphodiesterase I inhibitors based on an adenosine 5′-(α or γ)-thio-(α,β- or β,γ)-methylenetriphosphate scaffold. J. Med. Chem. 2014, 57, 4677–4691. 10.1021/jm500196c. 24846781 PMC4363092

[ref40] Graczyk A.; Pawlowska R.; Chworos A. Gold Nanoparticles as Carriers for Functional RNA Nanostructures. Bioconjugate Chem. 2021, 32, 1667–1674. 10.1021/acs.bioconjchem.1c00211. 34323473

[ref41] Madaj R.; Pawlowska R.; Chworos A. In silico exploration of binding of selected bisphosphonate derivatives to placental alkaline phosphatase via docking and molecular dynamics. J. Mol. Graphics Modell. 2021, 103, 107801 10.1016/j.jmgm.2020.107801. 33296741

[ref42] Madaj R.; Gostynski B.; Pawlowska R.; Chworos A. Tissue-Nonspecific Alkaline Phosphatase (TNAP) as the Enzyme Involved in the Degradation of Nucleotide Analogues in the Ligand Docking and Molecular Dynamics Approaches. Biomolecules 2021, 11 (8), 1104 10.3390/biom11081104. 34439771 PMC8391816

[ref43] Kukhanova M. K.; Zakirova N. F.; Ivanov A. V.; Alexandrova L. A.; Jasco M. V.; Khomutov A. R. Hypophosphoric acid is a unique substrate of pyrophosphorolysis catalyzed by HIV-1 reverse transcriptase. Biochem. Biophys. Res. Commun. 2005, 338, 1335–1341. 10.1016/j.bbrc.2005.10.092. 16271706

[ref44] Nycz J. E. The Synthesis of Hypodiphosphoric Acid and Derivatives with P-P Bond, including Esters and Diphosphine Dioxides: A Review. Molecules 2021, 26, 7286 10.3390/molecules26237286. 34885870 PMC8659023

[ref45] Stec W. J.; Grajkowski A.; Kobylańska A.; Karwowski B.; Koziołkiewicz M.; Misiura K.; Okruszek A.; Wilk A.; Guga P.; Boczkowska M. () Diastereomers of Nucleoside 3′-O-[2- Thio-1,3,2-oxathia(Selena)phospholanes]: Building Blocks for Stereocontrolled Synthesis of Oligo(nucleoside phosphorothioate)s. J. Am. Chem. Soc. 1995, 117, 12019–12029. 10.1021/ja00154a001.

[ref46] Guga P.; Stec W. J. Synthesis of phosphorothioate oligonucleotides with stereodefined phosphorothioate linkages. Curr. Protoc. Nucleic Acid Chem. 2003, 14, 4–17. 10.1002/0471142700.nc0417s14. 18428907

[ref47] Pawlowska R.; Janicka M.; Jedrzejczyk D.; Chworos A. RNA fragments mimicking tRNA analogs interact with cytochrome c. Mol. Biol. Rep. 2016, 43 (4), 295–304. 10.1007/s11033-016-3954-6. 26892782

