# Supplementary material for: Substrate Specificity of T7 RNA Polymerase toward Hypophosphoric Analogues of ATP
Source: ACS Omega. 2024 Feb 13;9(8):9348–56. doi: 10.1021/acsomega.3c08635 (PMC10905585; doi:10.1021/acsomega.3c08635)
Supplement: Supplementary file 1 — ao3c08635_si_001.pdf [file ao3c08635_si_001.pdf]

Supplementary Materials

Substrate specificity of T7 RNA polymerase towards hypophosphoric analogues of ATP

Roza Pawlowska[1]*, Anna Graczyk[1], Ewa Radzikowska-Cieciura[1], Ewelina Wielgus[2], Rafal Madaj[1], Arkadiusz Chworos[1]

[1]Department of Bioorganic Chemistry, Centre of Molecular and Macromolecular Studies, Polish Academy of Sciences, Sienkiewicza 112, 90-363 Lodz, Poland,

[2]Department of Structural Chemistry, Centre of Molecular and Macromolecular Studies, Polish Academy of Sciences, Sienkiewicza 112, 90-363 Lodz, Poland,

**Corresponding author: roza.pawlowska@cbmm.lodz.pl*

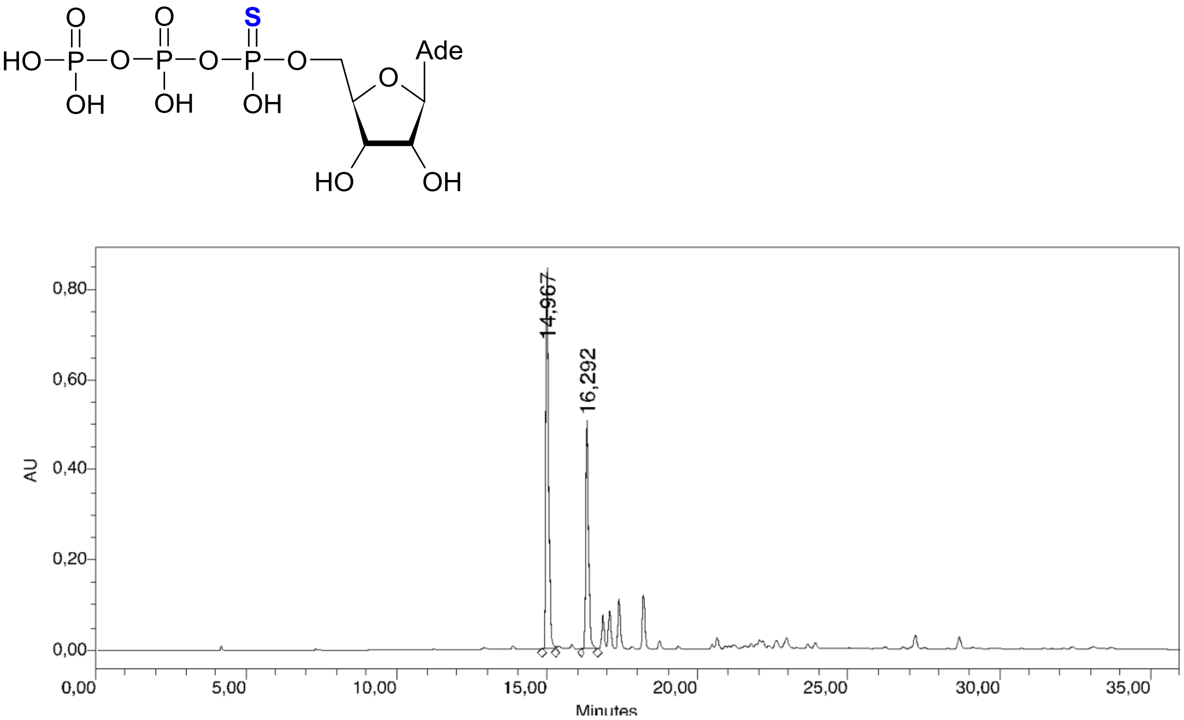

Figure S1. *HPLC profile of adenosine 5'-O-(Pα-thiotriphosphate) (**2**) after DEAE Sephadex purification.*

Single Mass Analysis
Tolerance = 5.0 PPM / DBE: min = -1.5, max = 90.0
Element prediction: Off
Number of isotope peaks used for i-FIT = 9

Monoisotopic Mass, Even Electron Ions
291 formula(e) evaluated with 1 results within limits (all results (up to 1000) for each mass)
Elements Used:
C: 0-15 H: 0-20 N: 0-6 O: 0-13 S: 1-1 P: 1-3

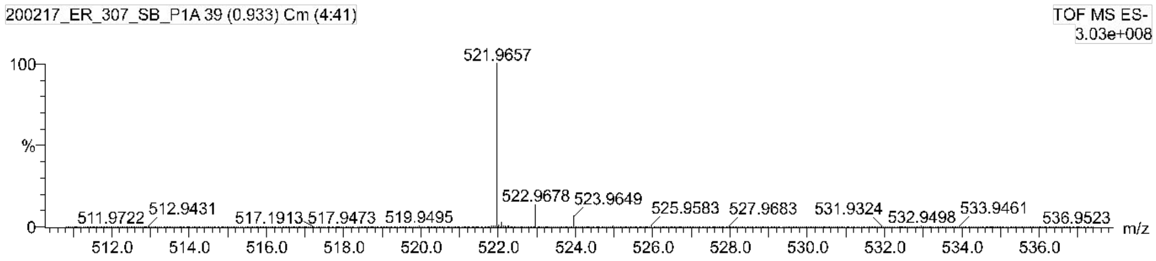

Minimum:			-1.5	
Maximum:	5.0	5.0	90.0	

Mass	Calc. Mass	mDa	PPM	DBE	i-FIT	Norm	Conf(%)	Formula
521.9657	521.9651	0.6	1.1	7.5	3797.3	n/a	n/a	C10 H15 N5 O12 S P3

Figure S2. *Mass spectrometry analysis of P-diastereoisomer* ***fast*** *of adenosine 5'-O-(Pα-thiotriphosphate) (S*P*-**2**) after HPLC purification.*

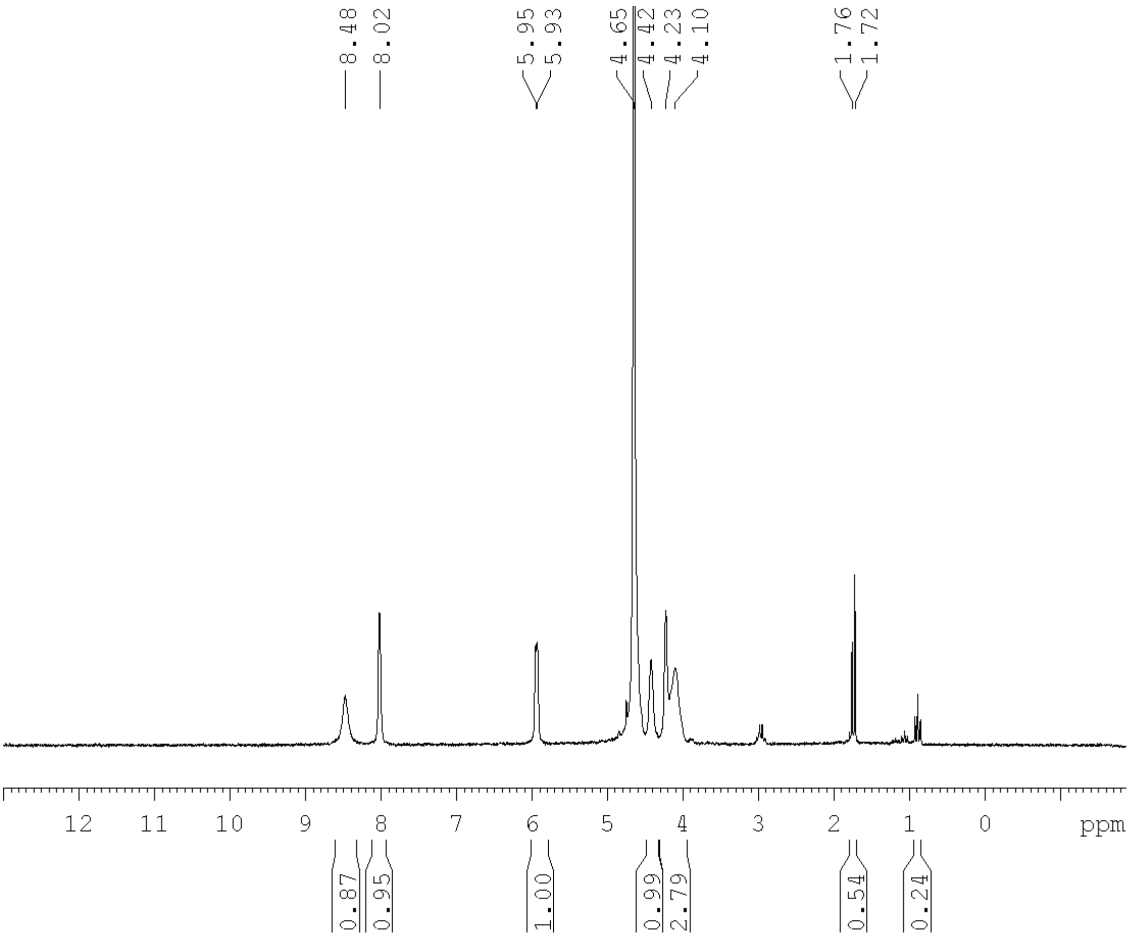

Figure S3**. ^{1}H NMR of P-diastereoisomer* ***fast *of adenosine 5'-O-(Pα-thiotriphosphate) (S*P*-**2**) after HPLC purification.*

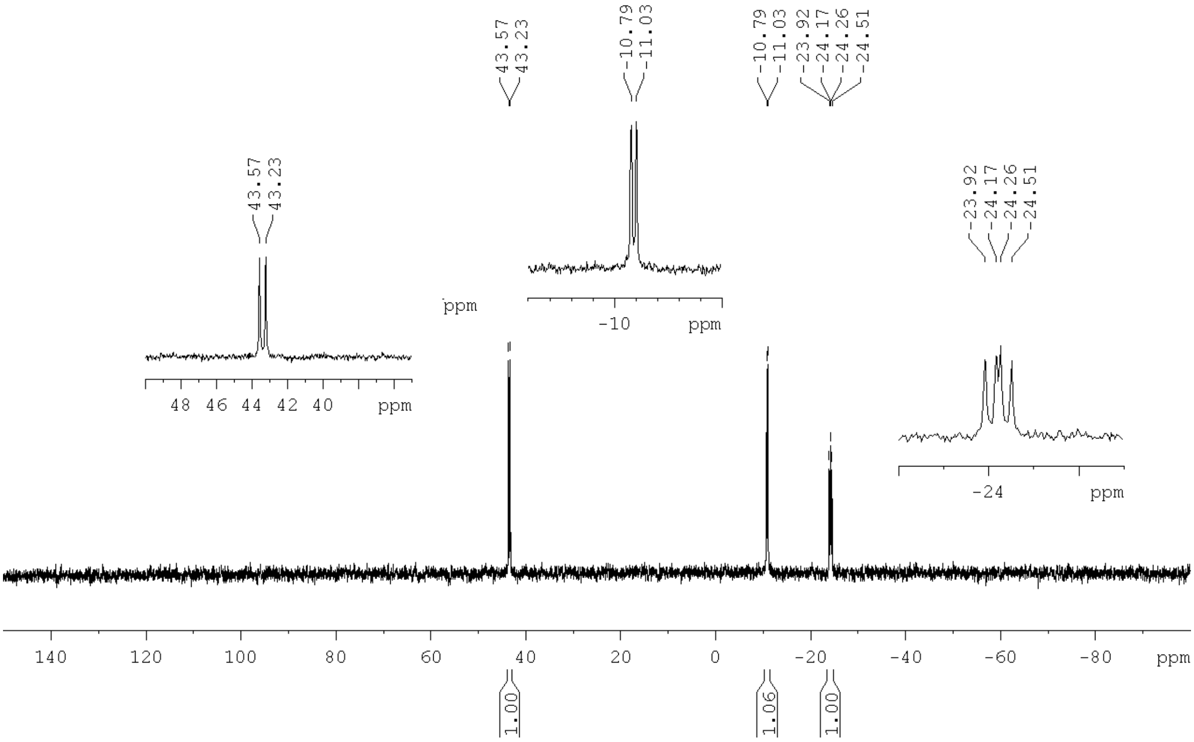

Figure S4. *^{31}P NMR of P-diastereoisomer* ***fast*** *of adenosine 5'-O-(Pα-thiotriphosphate) (S*P*-***2***) after HPLC purification.*

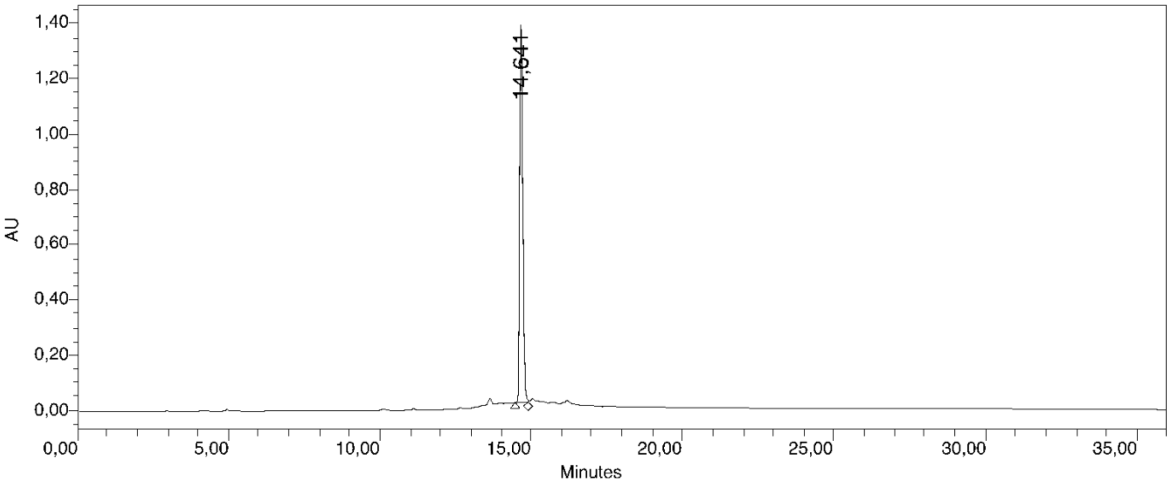

Figure S5. *HPLC profile of P-diastereoisomer* ***fast*** *of adenosine 5'-O-(Pα-thiotriphosphate) (S*P*-***2***).*

Single Mass Analysis

Tolerance = 5.0 PPM / DBE: min = -1.5, max = 90.0
Element prediction: Off
Number of isotope peaks used for i-FIT = 9

Monoisotopic Mass, Even Electron Ions
291 formula(e) evaluated with 1 results within limits (all results (up to 1000) for each mass)
Elements Used:
C: 0-15 H: 0-20 N: 0-6 O: 0-13 S: 1-1 P: 1-3

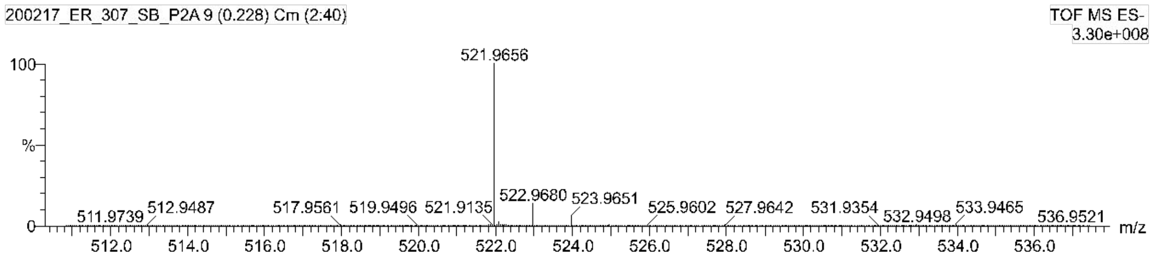

Minimum:				-1.5	
Maximum:		5.0	5.0	90.0	

Mass	Calc. Mass	mDa	PPM	DBE	i-FIT	Norm	Conf(%)	Formula
521.9656	521.9651	0.5	1.0	7.5	3552.4	n/a	n/a	C10 H15 N5 O12 S P3

Figure S6. *Mass analysis of P-diastereoisomer* **slow** *of adenosine 5'-O-(Pα-thiotriphosphate) (R*P*-***2***) after HPLC purification.*

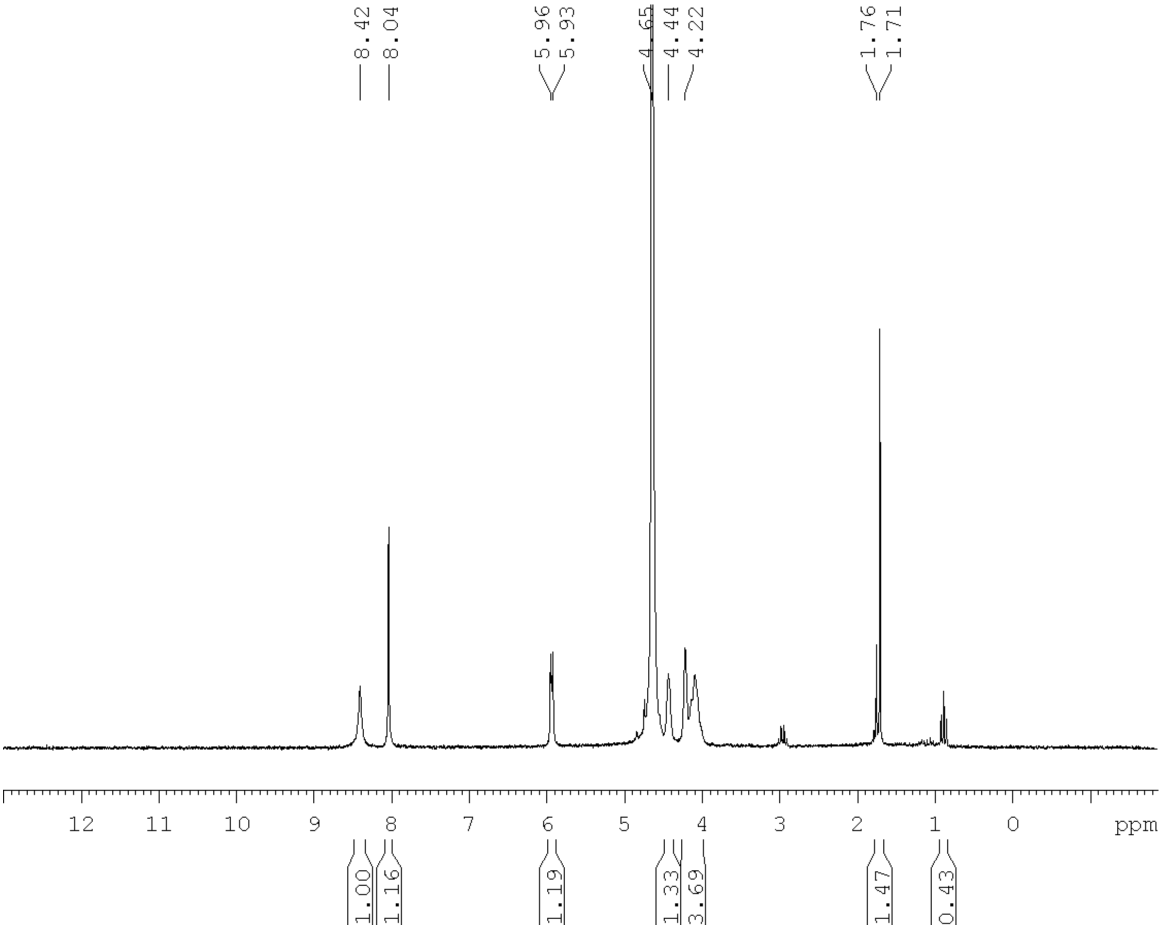

Figure S7. *^{1}H NMR of P-diastereoisomer* **slow** *of adenosine 5'-O-(Pα-thiotriphosphate) (R*P*-***2***) after HPLC purification.*

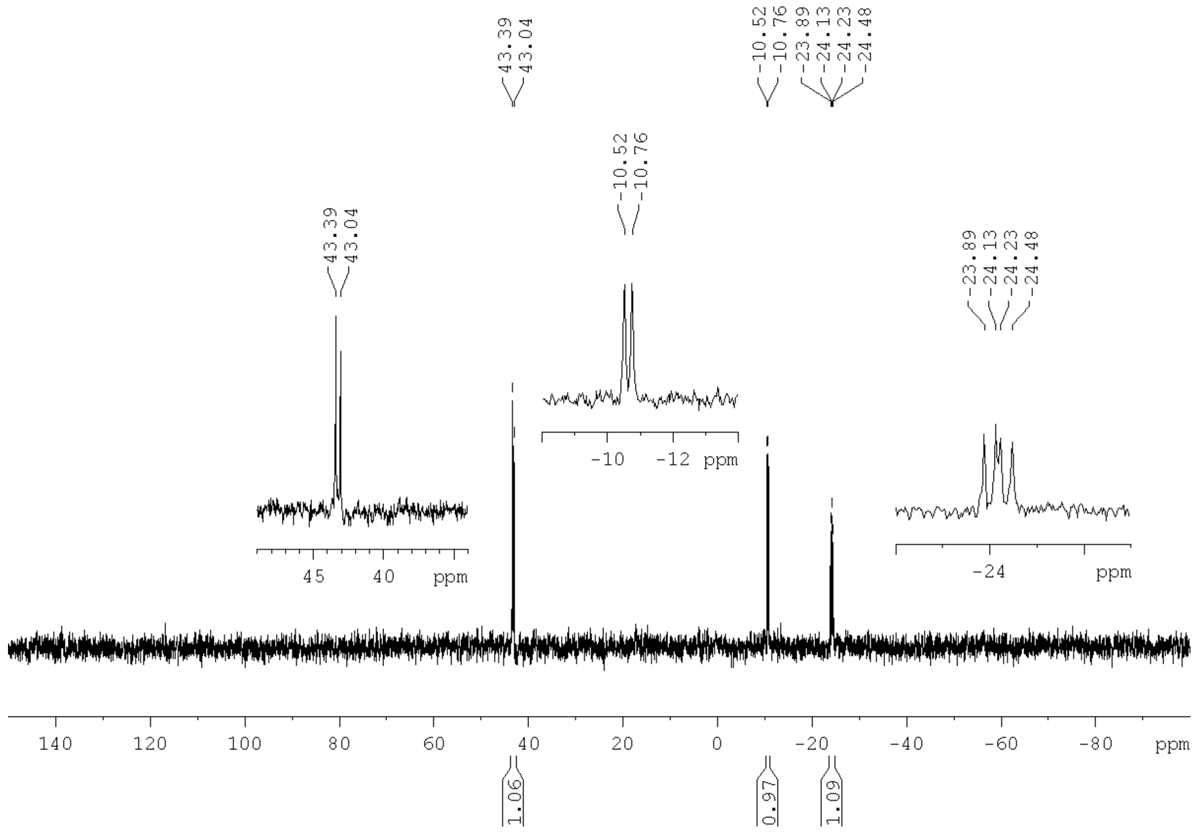

Figure S8. *^{31}P NMR of P-diastereoisomer* ***slow*** *of adenosine 5'-O-(Pα-thiotriphosphate) (R_P-**2**) after HPLC purification.*

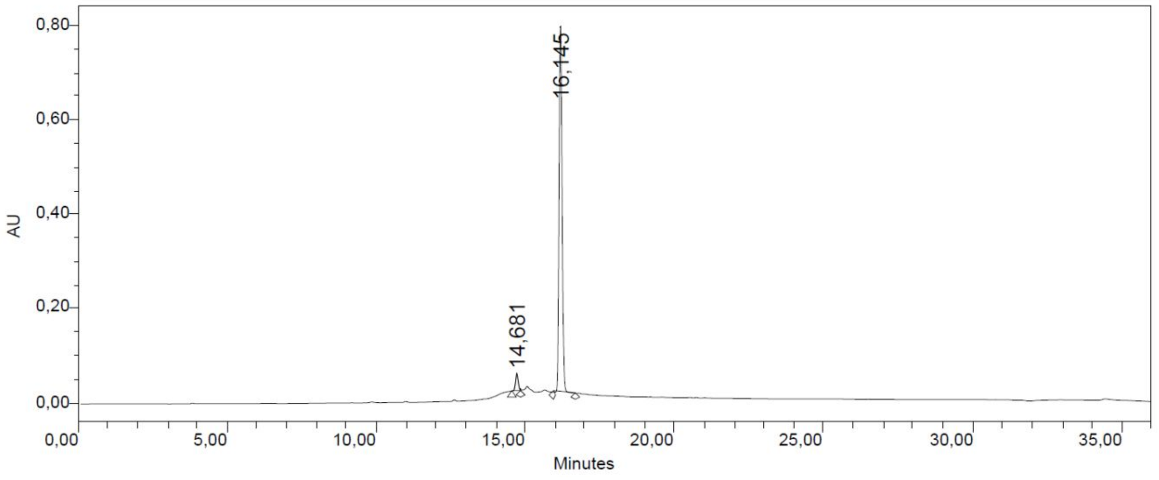

Figure S9. *HPLC profile of P-diastereoisomer* ***slow*** *of adenosine 5'-O-(Pα-thiotriphosphate) (R_P-**2**).*

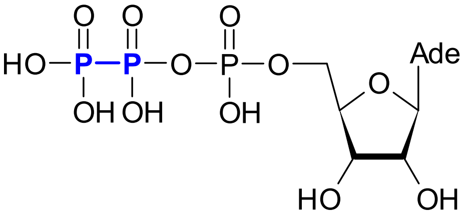

Single Mass Analysis
Tolerance = 5.0 PPM / DBE: min = -1.5, max = 70.0
Element prediction: Off
Number of isotope peaks used for i-FIT = 9

Monoisotopic Mass, Even Electron Ions
194 formula(e) evaluated with 3 results within limits (all results (up to 1000) for each mass)
Elements Used:
C: 0-20 H: 0-20 N: 0-5 O: 0-12 P: 3-3 S: 0-1
Radzikowska
191112_ER_304_p_glownyA 5 (0.141) Cm (4:40) TOF MS ES-
2.67e+005

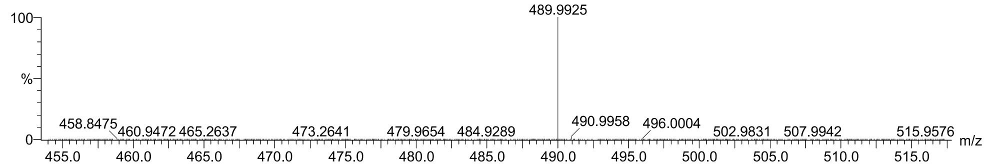

Mass	Calc. Mass	mDa	PPM	DBE	i-FIT	Norm	Conf(%)	Formula
Minimum:				-1.5				
Maximum:		5.0	5.0	70.0				
489.9925	489.9930	-0.5	-1.0	7.5	831.0	1.492	22.50	C10 H15 N5 O12 P3
	489.9945	-2.0	-4.1	15.5	830.5	0.957	38.40	C19 H15 N3 O5 P3 S
	489.9905	2.0	4.1	11.5	830.5	0.939	39.11	C14 H15 N5 O7 P3 S

Figure S10. *Mass analysis of P-diastereoisomer of adenosine 5'-O-(β,γ-hypotriphosphate) (3).*

Data for compound **3**: **^{1}H NMR** (D_2O, 200 MHz) δ: 8.47 (s, H-8, 1H), 8.12 (s, H-2, 1H), 5.96 (br. s, H-1′, 1H), 4.25-4.05 (m, H-2′, H-3′, H-4′ and 2xH-5′, 5H hidden in water signal), 2.98 (m, Et_3N traces), 1.07 (m, Et_3N traces) ppm; **^{31}P NMR** (D2O, 81 MHz) δ 4.50 (s, Pα, 1P), 3.21 (d, J = 26.2 Hz, Pβ, 1P), -10.56 (d, J = 25.0 Hz, Pβ, 1P) ppm; HRMS ESI (negative) *m/z* calculated for C11H15N5O11P3S $[M-H]^-$ − 489.9930, found 489.9925; The purity of compound was confirmed using RP HPLC analytical column: t_R = 13.2 min (97% purity)

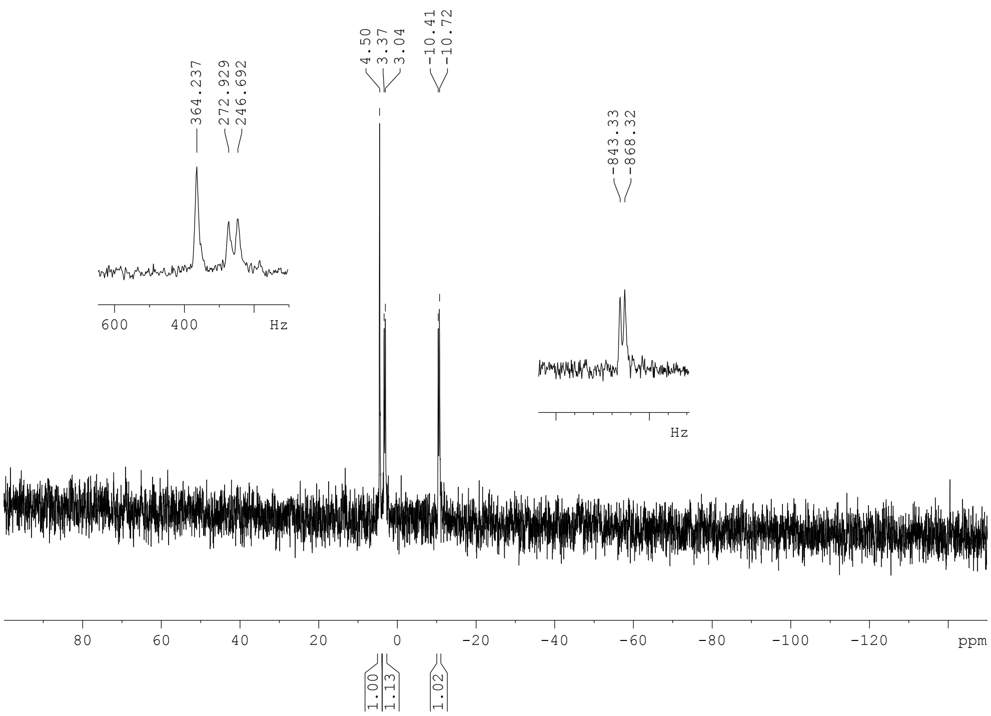

Figure S11. *^{31}P NMR of P-diastereoisomer of adenosine 5'-O-(β,γ-hypotriphosphate)* ***(3)*** *after HPLC purification.*

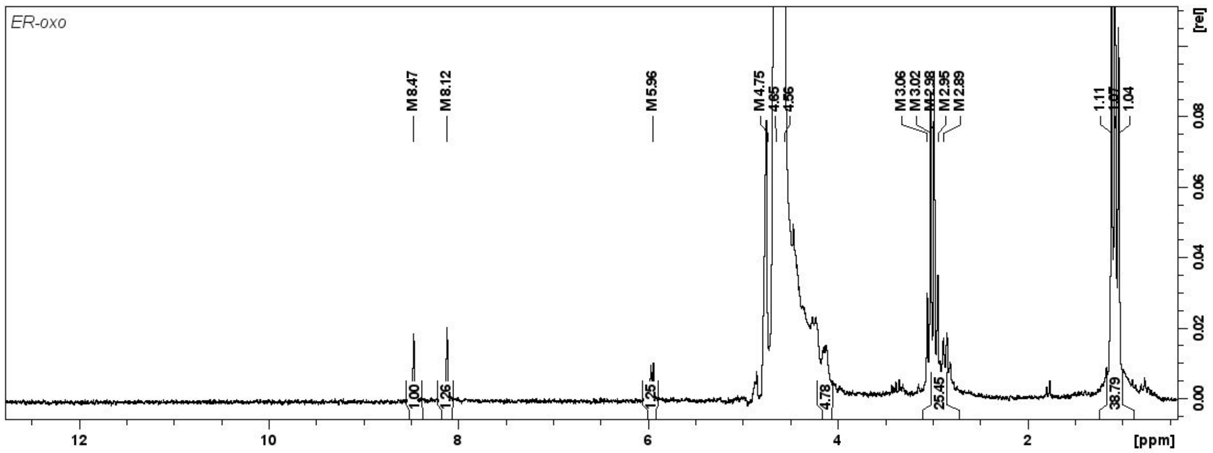

Figure S12. *^{1}H NMR of P-diastereoisomer of adenosine 5'-O-(β,γ-hypotriphosphate)* ***(3)*** *after HPLC purification.*

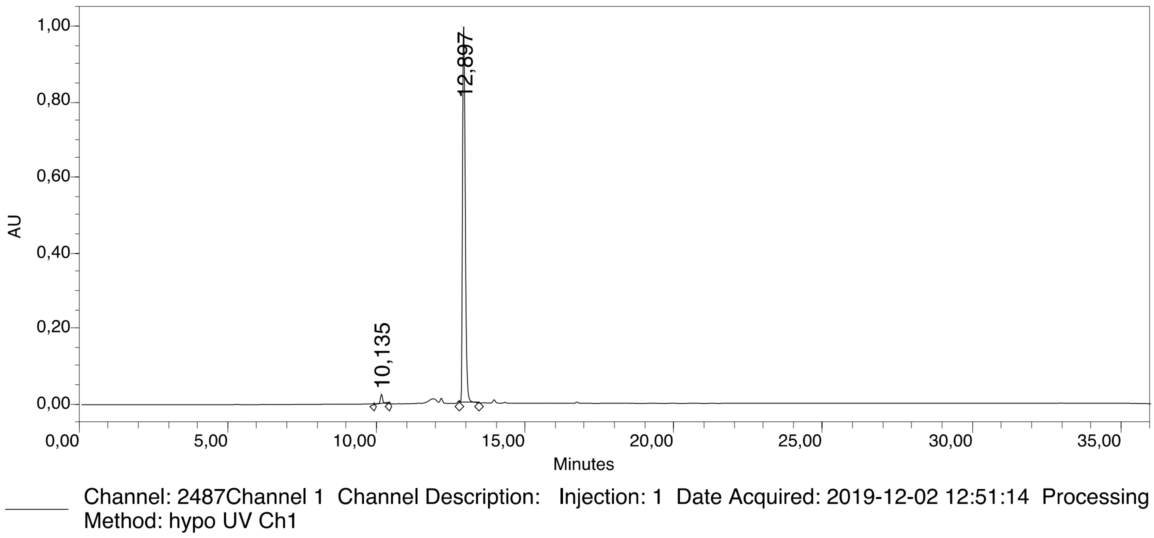

Figure S13. *HPLC profile of P-diastereoisomer of adenosine 5'-O-(β,γ-hypotriphosphate)* ***(3)****.*

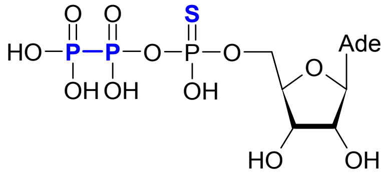

Single Mass Analysis
Tolerance = 5.0 PPM / DBE: min = -1.5, max = 80.0
Element prediction: Off
Number of isotope peaks used for i-FIT = 9

Monoisotopic Mass, Even Electron Ions
250 formula(e) evaluated with 1 results within limits (all results (up to 1000) for each mass)
Elements Used:
C: 0-20 H: 0-60 N: 0-6 O: 0-12 P: 3-3 S: 1-1
210212_ER_302_2_p2_FASTA 41 (0.437) Cm (38:50) TOF MS ES- 2.99e+007

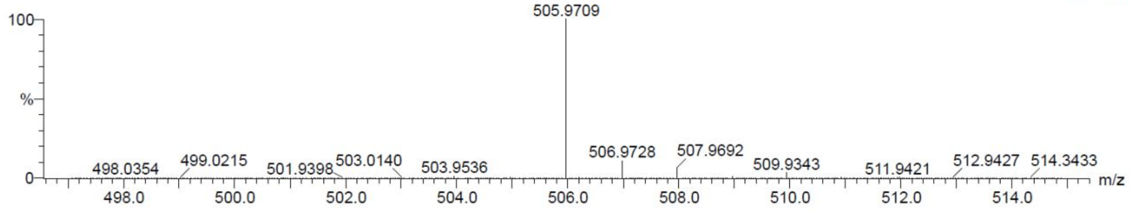

Minimum:				-1.5				
Maximum:		5.0	5.0	80.0				
Mass	Calc. Mass	mDa	PPM	DBE	i-FIT	Norm	Conf(%)	Formula
505.9709	505.9702	0.7	1.4	7.5	2286.8	n/a	n/a	C10 H15 N5 O11 P3 S

Figure S14. *Mass analysis of P-diastereoisomer* ***fast*** *of adenosine 5'-O-(α-thio-β,γ-hypotriphosphate) (S*P*-**4**) after HPLC purification.*

Data for isomer *S*P-**4**: **^{1}H NMR** (D_2O, 200 MHz) δ: 8.47 (s, H-8, 1H), 8.00 (s, H-2, 1H), 5.90 (d, J= 5.6 Hz, H-1′, 1H), 4.53 (m, H-2′, 1H), 4.39 (m, H-3′, 1H), 4.16 (m, H-4′, 2xH-5', 3H), 2.95 (m, CH_2, Et_3N, 18H), 1.03 (m, CH_3, Et3N, 27H) ppm; **^{31}P NMR** (D2O, 81 MHz) δ 43.69 (d, J = 32.9 Hz, Pα-S, 1P), 4.32 (s, Pγ, 1P), 2.97 (d, J = 33.0 Hz, Pβ, 1P) ppm; HRMS ESI (negative) *m/z* calculated for C11H15N5O11P3S $[M-H]^-$ − 505.9702, found 505.9709; The purity of compound was estimated using RP HPLC analytical column: t_R = 13.2 min (99.9% purity)

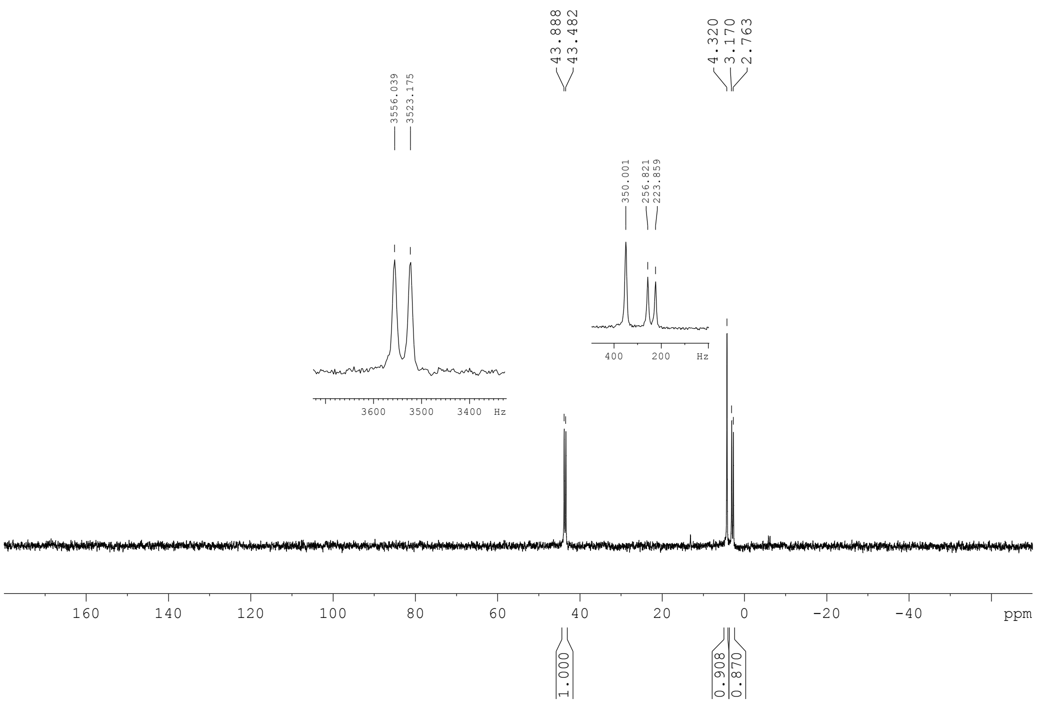

Figure S15. *^{31}P NMR of P-diastereoisomer* ***fast*** *of adenosine 5'-O-(α-thio-β,γ-hypotriphosphate) (S*P*-**4**) after HPLC purification.*

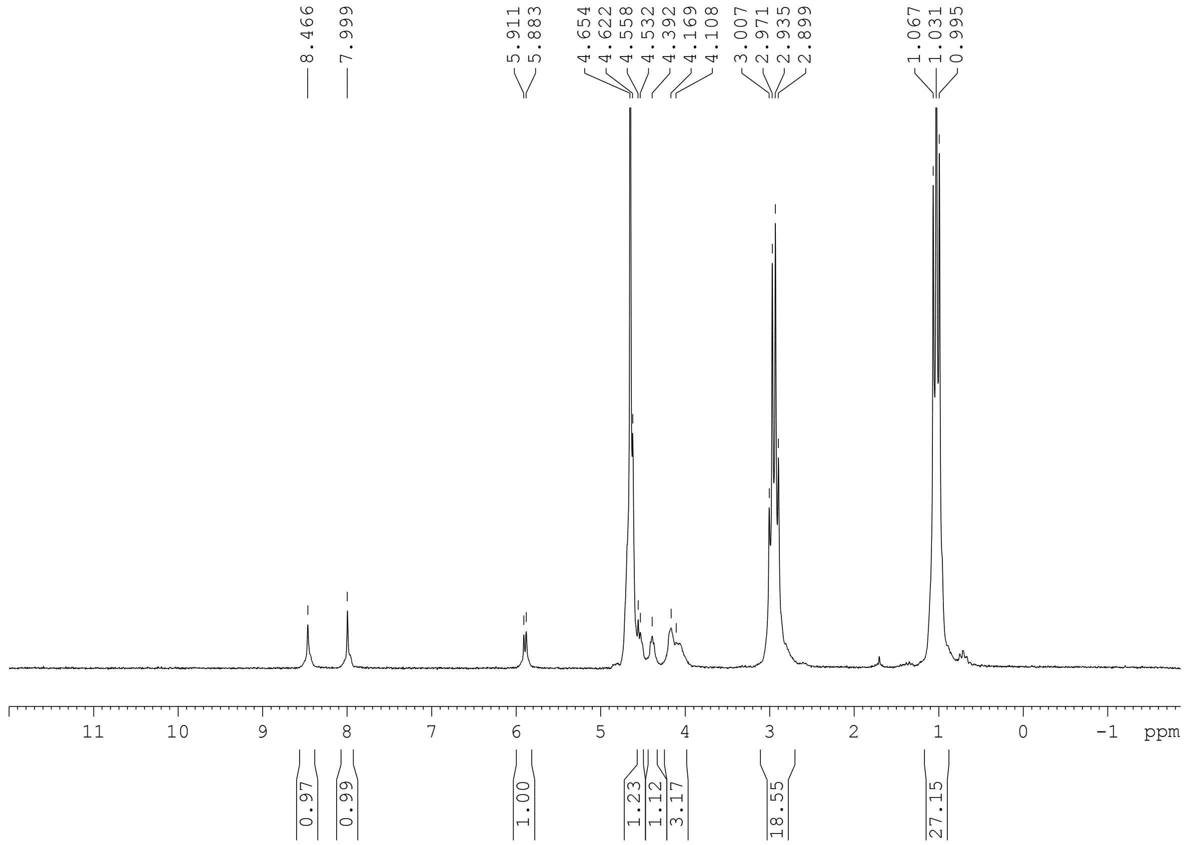

Figure S16. *^{1}H NMR of P-diastereoisomer* ***fast*** *of adenosine 5'-O-(α-thio-β,γ-hypotriphosphate)) (S*P*-**4**) after HPLC purification.*

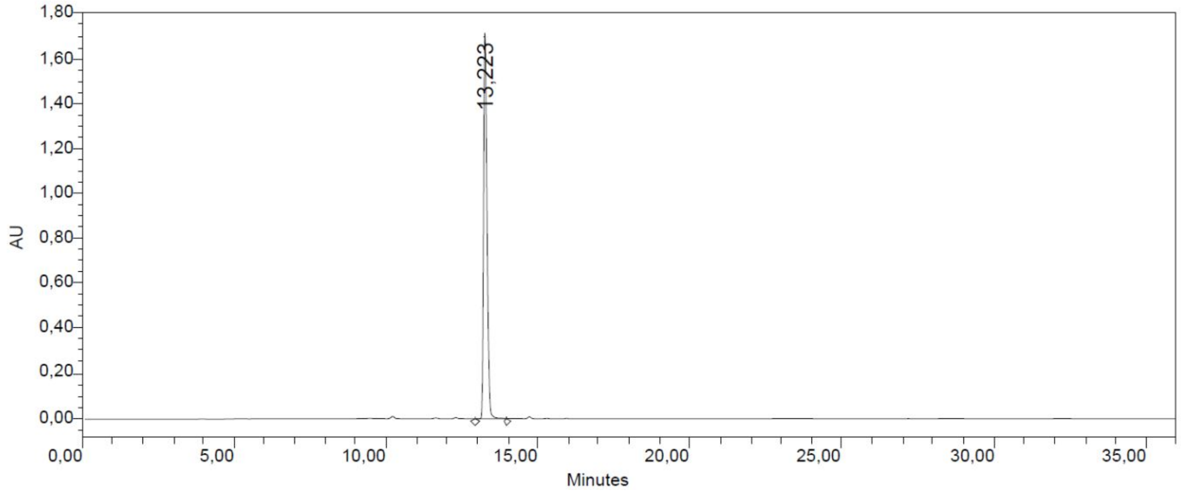

Figure S17. *HPLC profile of P-diastereoisomer* ***fast*** *of adenosine 5'-O-(α-thio-β,γ-hypotriphosphate) (S*$_P$*-****4****).*

Single Mass Analysis
Tolerance = 5.0 PPM / DBE: min = -1.5, max = 80.0
Element prediction: Off
Number of isotope peaks used for i-FIT = 9

Monoisotopic Mass, Even Electron Ions
250 formula(e) evaluated with 1 results within limits (all results (up to 1000) for each mass)
Elements Used:
C: 0-20 H: 0-60 N: 0-6 O: 0-12 P: 3-3 S: 1-1
210212_ER_302_2_p3_SLOW 65 (0.677) Cm (58:91)

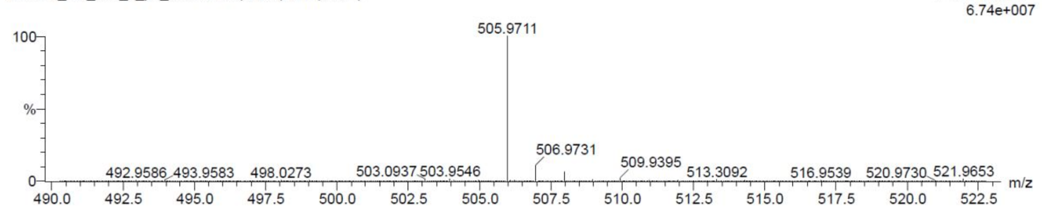

	Mass	Calc. Mass	mDa	PPM	DBE	i-FIT	Norm	Conf(%)	Formula
Minimum:					-1.5				
Maximum:			5.0	5.0	80.0				
	505.9711	505.9702	0.9	1.8	7.5	3626.6	n/a	n/a	C10 H15 N5 O11 P3 S

Figure S18. *Mass analysis of P-diastereoisomer* ***slow*** *of adenosine 5'-O-(α-thio-β,γ-hypotriphosphate) (R*$_P$*-****4****) after HPLC purification.*

Data for isomer *R*$_P$-**4**: **^{1}H NMR** (D_2O, 200 MHz) δ: 8.38 (s, H-8, 1H), 7.99 (s, H-2, 1H), 5.89 (d, J= 5.6 Hz, H-1′, 1H), 4.53 (m, H-2′, 1H), 4.39 (m, H-3′, 1H), 4.13 (m, H-4′, 2xH-5', 3H), 2.95 (m, CH_2, Et_3N, 18H), 1.03 (m, CH_3, Et3N, 27H) ppm; **^{31}P NMR** (D2O, 81 MHz) δ 43.46 (d, J = 33.3 Hz, Pα-S, 1P), 4.30 (s, Pγ, 1P), 3.02 (d, J = 33.0 Hz, Pβ, 1P) ppm; HRMS ESI (negative) *m/z* calculated for C11H15N5O11P3S [M-H]$^-$ − 505.9702, found 505.9711; The purity of compound was estimated using RP HPLC analytical column: t_R = 15.1 min (>99% purity)

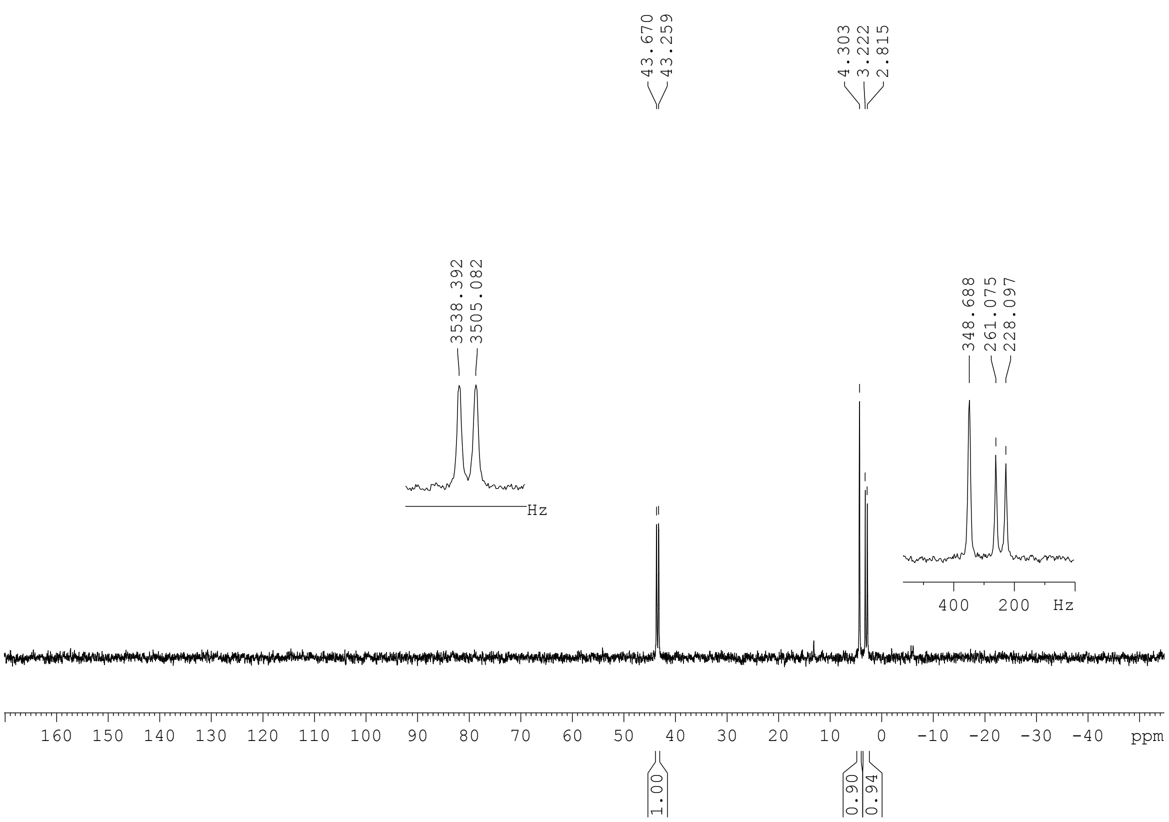

Figure S19. *^{1}H NMR of P-diastereoisomer* ***slow*** *of adenosine 5'-O-(α-thio-β,γ-hypotriphosphate) (R*$_P$*-**4**) after HPLC purification.*

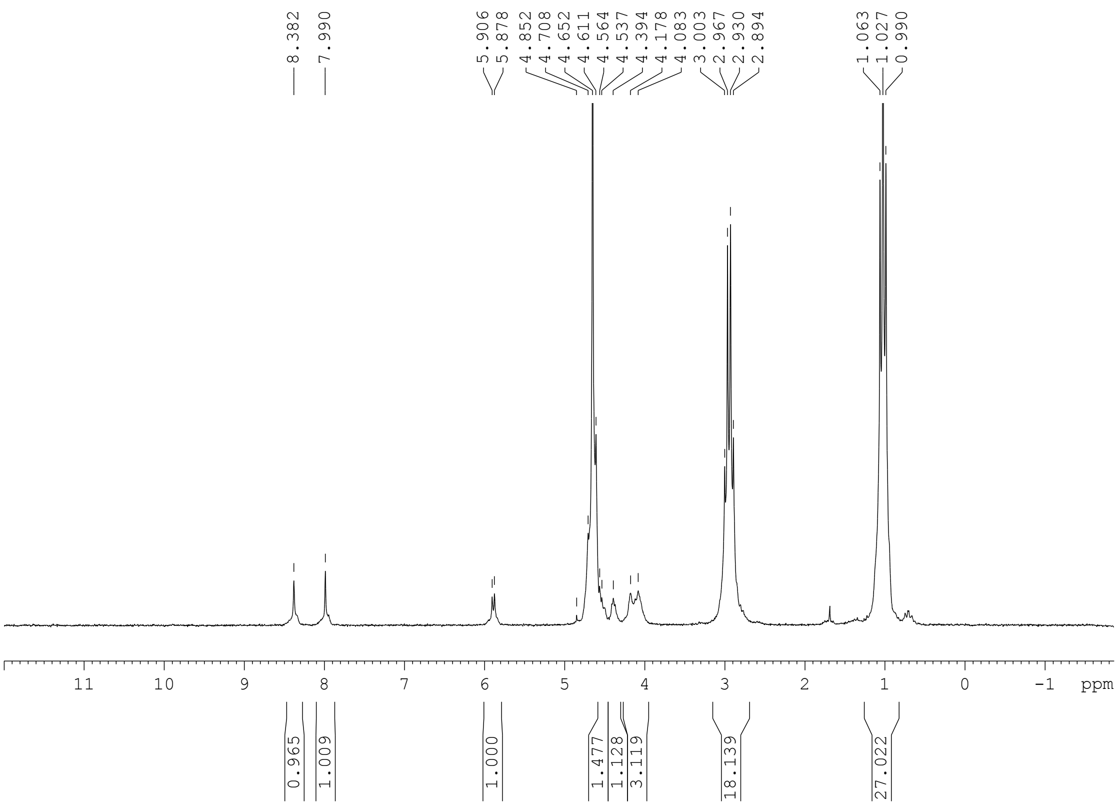

Figure S20. *^{1}H NMR of P-diastereoisomer* ***slow*** *of adenosine 5'-O-(α-thio-β,γ-hypotriphosphate) (R*$_P$*-**4**) after HPLC purification.*

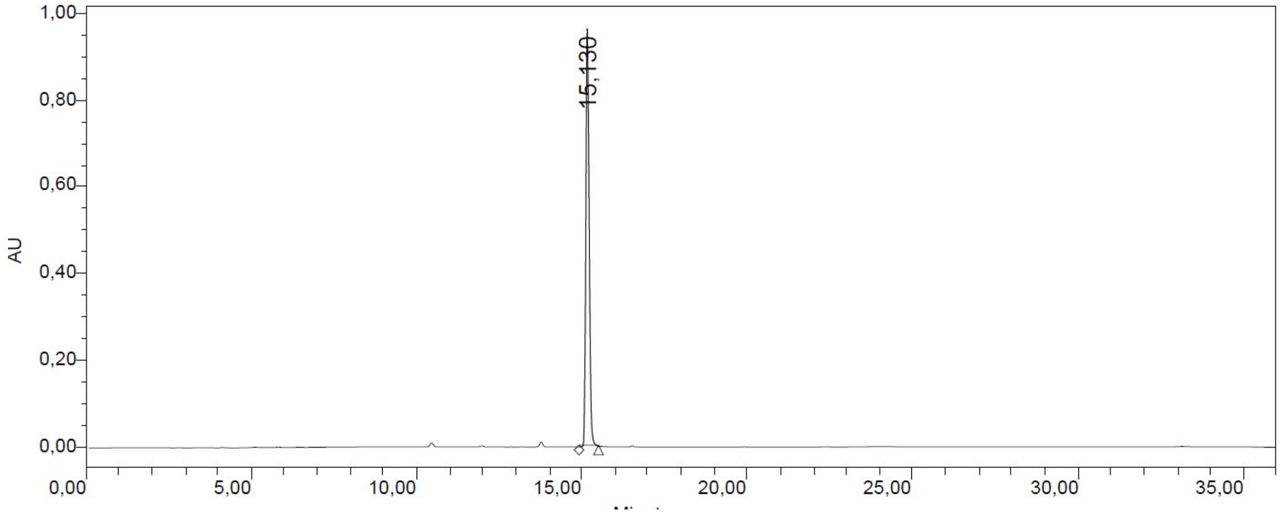

Figure S21. *HPLC profile of P-diastereoisomer* ***slow*** *of adenosine 5'-O-(α-thio-β,γ-hypotriphosphate) (*R_P*-**4**).*

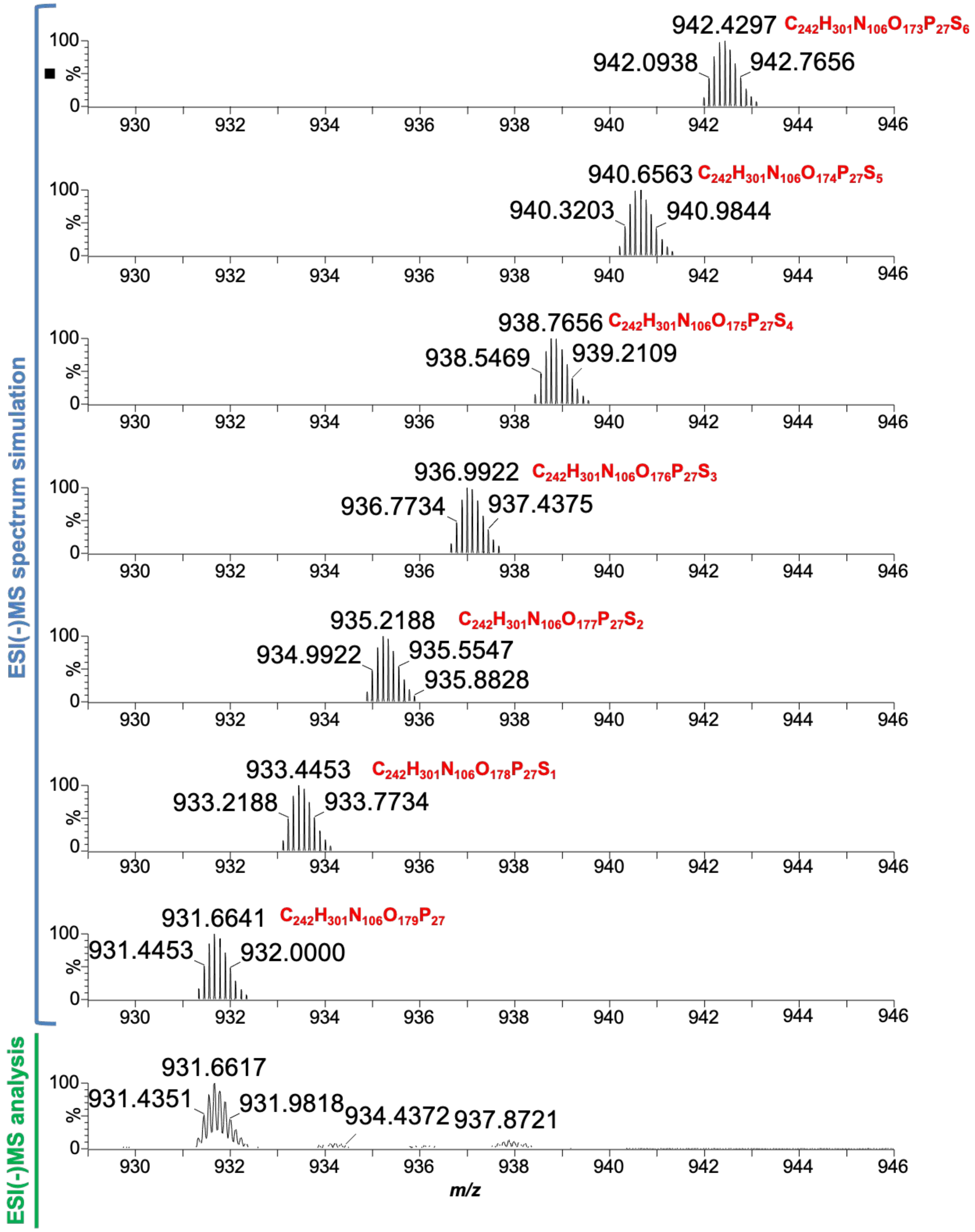
ESI(-)MS spectrum simulation
ESI(-)MS analysis
942.4297 $C_{242}H_{301}N_{106}O_{173}P_{27}S_6$
942.0938
942.7656
940.6563 $C_{242}H_{301}N_{106}O_{174}P_{27}S_5$
940.3203
940.9844
938.7656 $C_{242}H_{301}N_{106}O_{175}P_{27}S_4$
938.5469
939.2109
936.9922 $C_{242}H_{301}N_{106}O_{176}P_{27}S_3$
936.7734
937.4375
935.2188 $C_{242}H_{301}N_{106}O_{177}P_{27}S_2$
934.9922
935.5547
935.8828
933.4453 $C_{242}H_{301}N_{106}O_{178}P_{27}S_1$
933.2188
933.7734
931.6641 $C_{242}H_{301}N_{106}O_{179}P_{27}$
931.4453
932.0000
931.6617
931.4351
931.9818
934.4372
937.8721
100
%
0
930
932
934
936
938
940
942
944
946
m/z

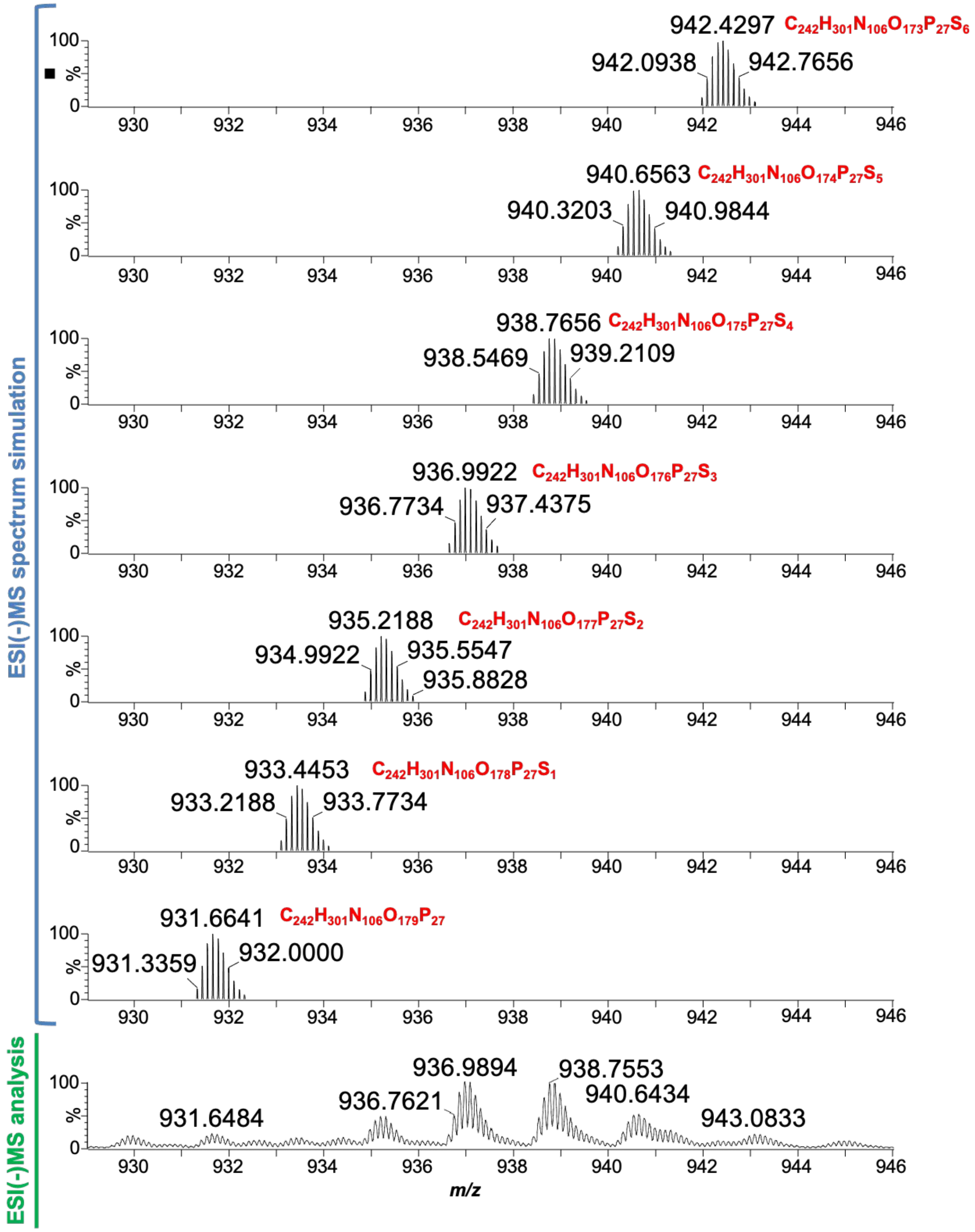

Figure S22. *Mass spectrometry analysis of the [M-9H⁺]⁹⁻ ion of the reaction products of the in vitro transcription conducted in the presence of ATP and α-thio-modified ATP analogues: ATP with S*$_P$*-**4** (upper panel) or in presence of S*$_P$*-**2** with ATP (lower panel). From the top – simulated mass spectra of unmodified and PS-modified products containing from 1 to 6 phosphorothioate modifications, on the bottom – final products of transcription reactions.*

Table S1. *Calculated and experimentally confirmed monoisotopic mass of RNA strands synthetized by the T7 RNA polymerase in the presence of ATP and α-thio-modified ATP analogues*

**Number of PS modifications in the synthesized RNA strand**	**Elementary content**	**Calculated monoisotopic mass**	**Experimentally confirmed monoisotopic mass**
**6**	C242H301N106O173P27S6	**8486.93**	**8486.84**
**5**	C242H301N106O174P27S5	**8470.95**	**8470.81**
**4**	C242H301N106O175P27S4	**8454.97**	**8454.87**
**3**	C242H301N106O176P27S3	**8438.99**	**8438.88**
**2**	C242H301N106O177P27S2	**8423.02**	**8422.94**
**1**	C242H301N106O178P27S1	**8407.04**	**8406.93**
**0**	C242H301N106O179P27	**8391.06**	**8390.97**

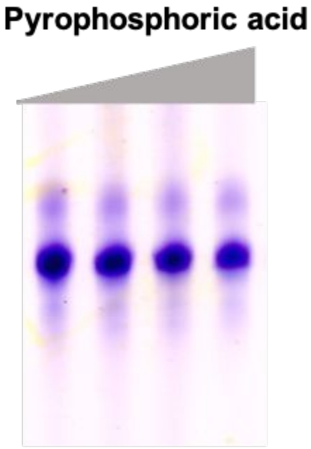

Figure S23. *PAGE analysis of the in vitro transcription efficiency in the present of the increasing amount of pyrophosphoric acid: 0.1, 0.5, 1 and 2 mM, respectively.*
